# Structural Characterization of Nanoparticle-Supported Lipid Bilayer Arrays by Grazing Incidence X-ray and Neutron Scattering

**DOI:** 10.1021/acsami.2c18956

**Published:** 2023-01-10

**Authors:** Nicolò Paracini, Philipp Gutfreund, Rebecca Welbourn, Juan Francisco Gonzalez-Martinez, Kexin Zhu, Yansong Miao, Nageshwar Yepuri, Tamim A. Darwish, Christopher Garvey, Sarah Waldie, Johan Larsson, Max Wolff, Marité Cárdenas

**Affiliations:** † Department for Biomedical Science and Biofilms − Research Center for Biointerfaces, Faculty of Health and Society, Malmö University, 205 06Malmö, Sweden; ‡ Institut Laue-Langevin (ILL), 38000Grenoble, France; § ISIS Neutron & Muon Source, STFC, Rutherford Appleton Laboratory, Harwell, OxfordshireOX11 0QX, U.K.; ∥ School of Biological Sciences, Nanyang Technological University, 639798Singapore; ⊥ National Deuteration Facility, Australian Nuclear Science and Technology Organization (ANSTO), Lucas Heights, NSW2234, Australia; # Heinz Maier-Leibnitz Zentrum (MLZ), Technische Universität München, Lichtenbergstraβe 1, 85748Garching, Germany; ∇ Department of Physics and Astronomy, Uppsala University, Box 516, 751 20Uppsala, Sweden

**Keywords:** nanoparticle-supported lipid bilayers, nanoSLB, membrane curvature, model membranes, neutron reflectometry, GISANS, GISAXS

## Abstract

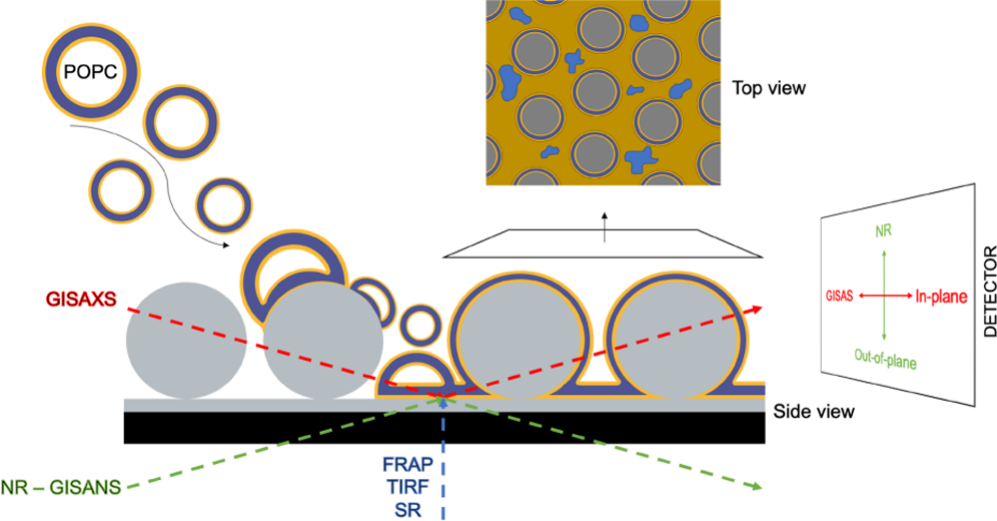

Arrays of nanoparticle-supported lipid bilayers (nanoSLB) are lipid-coated nanopatterned interfaces that provide a platform to study curved model biological membranes using surface-sensitive techniques. We combined scattering techniques with direct imaging, to gain access to sub-nanometer scale structural information on stable nanoparticle monolayers assembled on silicon crystals in a noncovalent manner using a Langmuir–Schaefer deposition. The structure of supported lipid bilayers formed on the nanoparticle arrays via vesicle fusion was investigated using a combination of grazing incidence X-ray and neutron scattering techniques complemented by fluorescence microscopy imaging. Ordered nanoparticle assemblies were shown to be suitable and stable substrates for the formation of curved and fluid lipid bilayers that retained lateral mobility, as shown by fluorescence recovery after photobleaching and quartz crystal microbalance measurements. Neutron reflectometry revealed the formation of high-coverage lipid bilayers around the spherical particles together with a flat lipid bilayer on the substrate below the nanoparticles. The presence of coexisting flat and curved supported lipid bilayers on the same substrate, combined with the sub-nanometer accuracy and isotopic sensitivity of grazing incidence neutron scattering, provides a promising novel approach to investigate curvature-dependent membrane phenomena on supported lipid bilayers.

## Introduction

Supported lipid bilayers (SLBs) are robust and widespread models of biological membranes with applications ranging from biophysical studies of membrane function^1^ to biosensing.^2^ Planar SLBs typically consist of phospholipid bilayers formed via vesicle fusion,^3^ solvent-assisted bilayer formation,^4^ or Langmuir–Blodgett and Langmuir–Schaefer monolayer transfer techniques^5,6^ onto flat hydrophilic interfaces of materials like quartz, mica, and silicon oxide. The wide variety of model cell membranes available for biophysical research offer a plethora of possible approaches to study the different aspects of membrane bioscience that are often too complex to address directly on the natural cell envelope, such as lipid–lipid and protein–lipid interactions.^1^ The effect of membrane morphology on the functional and structural properties of the lipid and protein components of plasma membranes is one of the aspects that remains more elusive, partly due to the scarcity of suitable model membrane systems. Membrane curvature has been shown to affect lipid and protein sorting and segregation^7,8^ in processes that contribute to vital functions of cells such as cell division and membrane remodeling.^9−11^ A well-characterized example of curvature-dependent lipid segregation is the preferential partition of the cone-shaped lipid cardiolipin into regions of negative curvature such as those found at the polar regions of rod-shaped bacteria^12,13^ and in the lumen-facing leaflet of mitochondrial cristae.^14^ Experiments on model lipid bilayers have shown that when cardiolipin is incorporated into large unilamellar vesicles of 100 nm in diameter, cardiolipin molecules display a 4:1 preference for the inner leaflet of the liposomes,^15^ and this degree of curvature is in turn able to modulate cardiolipin binding to cytochrome c.^16^ Most curvature-dependent lipid segregation studies rely on fluorescence microscopy and the use of fluorophores which are either covalently linked to or preferentially interact with target lipid molecules to track and visualize. Lipids preference for a certain type of curvature is highly dependent on molecular shape, which can easily be altered by the steric hindrance of covalently linked fluorescent probes while the use of added fluorescent stains has led to debates around their target specificity in membrane curvature studies.^17^ Deuterium labeling, in combination with isotope-sensitive neutron scattering techniques, represents a promising and complementary alternative to fluorescent labels that largely preserves the molecular shape of lipids and enables structural studies with sub-nanometer accuracy of model membranes with complex morphologies.

There is growing interest toward the development of nonplanar SLBs that deviate from canonical flat interfaces and instead display a degree of curvature of the lipid bilayer, recreating morphological aspects of the cell surface, which can display a wide range of degrees of curvature. These model systems find applications in the study of membrane curvature-mediated phenomena such as lipid and protein segregation and binding of curvature-sensitive proteins.^7,18−23^ For this purpose, nano- and micropatterned surfaces represent attractive substrates for the formation of SLBs with a well-defined surface morphology imparted by the underlying interfacial nanostructure. Both top-down and bottom-up methods have been adopted for the modification of flat interfaces and the formation of surface patterns suitable to form curved SLBs. Several top-down approaches rely on nanolithography techniques, which afford a high degree of control and fine-tuning over the resulting surface structure.^7,19^ The high precision of top-down methods however often comes at the cost of time and resources, which can become limiting factors when dealing with large surfaces and number of substrates to functionalize. Bottom-up approaches, on the other hand, typically rely on self-assembly processes driven by chemical and physical forces and can be exploited to fabricate patterned samples using nanoparticles (NP) to serve as substrates for SLB formation.^18,20,24^ Among bottom-up methods employed to form large arrays of NPs, Langmuir–Blodgett and Langmuir–Schaefer depositions offer an additional level of control on the self-assembly process by enabling the adjustment of the packing density of the Langmuir monolayer at the air/water interface prior to its transfer onto a solid substrate.^25−27^ Furthermore, Langmuir transfer techniques have the advantage of yielding large uniform monolayers, which make well-suited samples for characterization by flux-limited grazing incidence scattering methods such as neutron reflectometry (NR) and grazing incidence neutron small-angle scattering (GISANS).^28,29^ Due to their unique ability to probe noninvasively buried interfaces and their differential sensitivity toward hydrogen and deuterium, neutrons are among the most powerful surface-sensitive techniques for the structural characterization of complex biological thin films at solid/liquid interfaces, of which SLBs represent a primary example. Grazing incidence neutron scattering, particularly NR, has found wide application in the structural characterization of planar SLBs; however, the potential of techniques like GISANS, as well as NR, remains largely untapped when it comes to structural studies of model membranes with a more complex morphology. In this context, NR and GISANS, combined with selective lipid deuteration, can provide a novel approach to study the effect of curvature on the in-plane and out-of-plane structural features of SLBs.

In this article, we exploit a modified Langmuir–Schaefer deposition method to form large arrays of spherical silica NP on silicon oxide surfaces that are used as substrates to form nanoparticle-supported lipid bilayers (nanoSLB). First, we combine grazing incidence X-ray and neutron scattering to probe the structure of the NP arrays in both dry and aqueous environments (i.e., at the solid/air and solid/liquid interface). The formation of lipid bilayers via vesicle fusion on the nanoparticle arrays, investigated by quartz crystal microbalance with dissipation (QCMD) and fluorescence microscopy, indicates that lipids fuse on the substrate to form a lipid bilayer that retains lateral mobility. Finally, the in-plane and out-of-plane structural features of the nanoSLB system are resolved by NR and corroborated by GISANS revealing the formation of both a curved lipid bilayer coating the spherical particles as well as a planar bilayer on the underlying flat substrate, highlighting the coexistence of flat and curved regions.

## Results and Discussion

**Figure 1:**
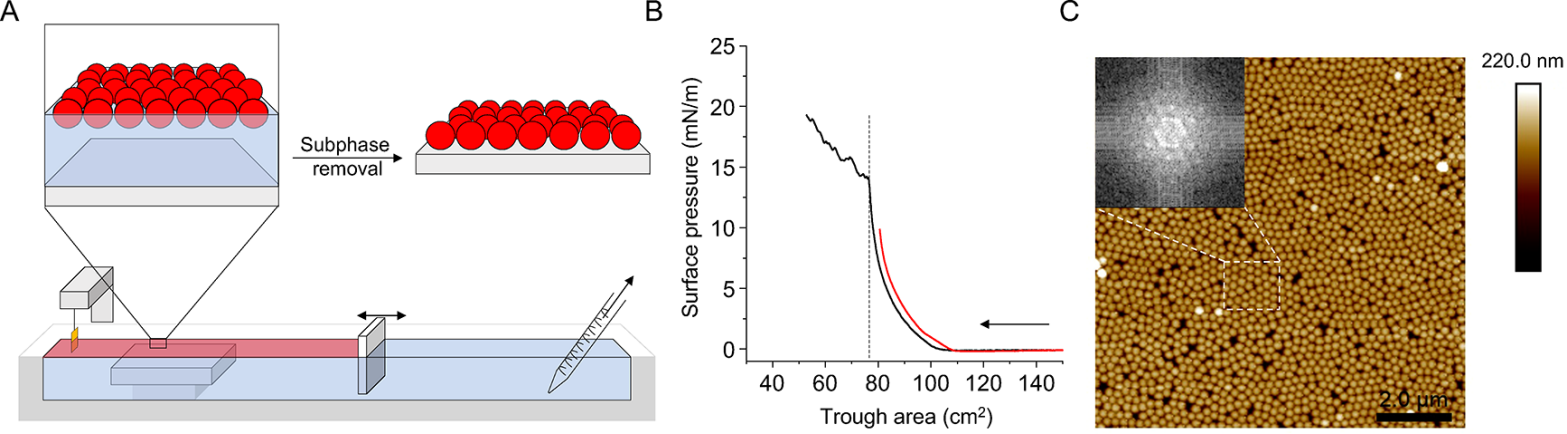
Langmuir–Schaefer transfer of a NP monolayer onto a solid substrate. (A) Schematic representation of the modified Langmuir–Schaefer transfer of the silica NP monolayer onto a submerged silicon crystal. Once the NP monolayer (red area) was compressed to the target surface pressure, measured by the Wilhelmy plate, the aqueous subphase was slowly removed from behind the barrier using a serological pipette tip connected to a pump. (B) Pressure–area isotherm of NP monolayer compressed above (black) and below (red) the collapse point at 15 mN/m and indicated by an abrupt change in the slope (dashed line); the arrow indicates the direction of compression. (C) NP monolayer (nominal diameter 2000 Å) after transfer onto a silicon wafer imaged by AFM. Black areas correspond to gaps between the particles, while bright spots are particles absorbed on top of the NP monolayer. The scale bar is 2 μm. The inset shows a Fourier transform of the highlighted region in the AFM image and the corresponding hexagonal lattice.

The formation of densely packed arrays of nonporous silica NPs was achieved via a modified Langmuir–Schaefer transfer of NPs from the air/water interface onto a pre-submerged polished silicon substrate,^30,31^ as depicted in Figure 1A. To maximize the particle density in the monolayer, pressure–area isotherms were recorded to establish the maximum surface pressure applicable to the NP monolayer at the air–water interface before excessive compression resulted in a collapse, indicated by an abrupt change in surface pressure. A value slightly below the collapse pressure was selected for NP depositions (Figure 1B). Atomic force microscopy (AFM) confirmed the formation of an NP layer with a high density of particles packed in a hexagonal lattice and interspersed with minor defects (Figure 1C).

Imaging techniques, such as AFM, only provide local information over small regions. Scattering methods, on the other hand, can probe the average structure over the large areas obtained using the Langmuir–Schaefer assembly method, providing information on the structure of complex thin films in both dry and aqueous environments.

### X-ray and Neutron Characterization of NP Monolayers

**Figure 2:**
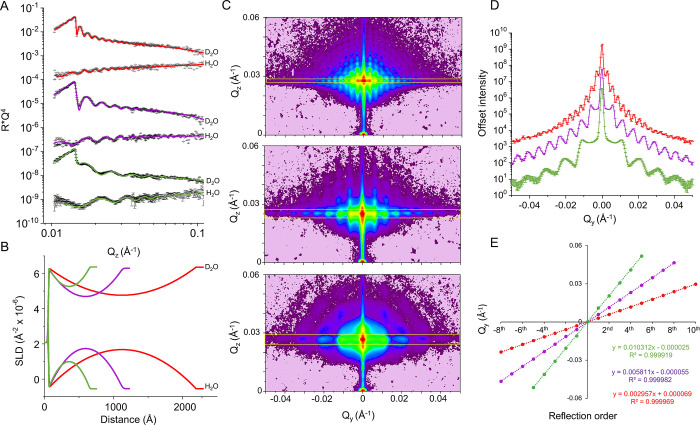
Structural characterization of NP monolayers by NR and GISAXS (A) Neutron reflectometry data (points) and best fit (lines) of three monolayers containing NP of different sizes, measured in D_2_O and H_2_O. Data and corresponding fits are offset vertically for clarity. (B) Corresponding SLD profile derived from the constrained fit of NR data from the two solution isotopic contrasts for each NP set. (C) GISAXS detector images of three monolayers measured at the air/solid interface. (D) Plot of the integrated intensities within the yellow boxes shown in (C). Absolute intensity is offset for clarity. (E) Linear regressions of the maxima positions along *Q*_y_ of the peaks shown in (D) displaying the equations of the fits and the *R*^2^ values. The *x* coefficient in the equations corresponds to the average separation of the peaks, Δ*Q*_y_.

Following the assembly method illustrated in Figure 1, monolayers of commercial NP with nominal diameters of 50, 100, and 200 nm were prepared and characterized by NR and GISAXS at the solid/liquid and air/solid interfaces (Figure 2). The samples, assembled into custom-built solid/liquid cells, were characterized by NR in an aqueous environment. NR measurements enable the determination of the structure of buried thin films along the axis perpendicular to the substrate, yielding a profile describing the scattering length density (SLD) distribution, which determines the neutron refractive index along the normal to the surface. Reflectivity curves measured in the presence of H_2_O and D_2_O were fitted simultaneously to a model of the interface describing a monolayer of spheres as detailed in the Supporting Information and Figure S1. The SLD profiles obtained from the constrained fits to the reflectivity curves collected in H_2_O and D_2_O yielded NP monolayer thicknesses of ∼2146, ∼1088, and ∼602 Å for the three samples (Table S1), in good agreement with the nominal size of the particles and the sizes obtained from dynamic light scattering (DLS) measurements (Figure S2), confirming the formation of NP monolayers (Figures 2A and S3). The in-plane packing density of the particles in the monolayers was extracted from the contrast provided by the hydrogenous and deuterated aqueous solutions and was found to be ∼66% for the largest particles, ∼61% for the intermediate size, and ∼42% for the smallest NPs (Table S1), where a value of 100% in the model corresponds to a defect-free layer of spheres ideally packed in a hexagonal lattice. The significantly worse packing of the smallest particles is indicative of larger areas occupied by defects in the NP monolayer, which might result from a size-dependent tendency to form inhomogeneous aggregates during the drying step following the Langmuir–Schaefer procedure. DLS showed a higher polydispersity for the smallest particles compared to the larger 100 and 200 nm NPs, which might have contributed to hindering ideal hexagonal packing; however, even for the 50 nm particles, polydispersity index remained below 0.1, indicating a highly monodisperse population.

To complement the structural information obtained by NR, the NP arrays were investigated by GISAXS, which is sensitive to the in-plane arrangement and correlations between the NPs.^32^ All three particle sizes gave rise to a strong GISAXS signal displaying several orders of well-defined peaks resulting from the scattering produced by the particle arrays (Figure 2C). The intensity and Q spacing of the peaks encodes information on the size and shape of the particles in the array, according to the inverse relationship between the *Q*_y_ spacing of the maxima and the corresponding real-space structures that generated them, according to *d* = 2π/Δ*Q*_y_. The average in-plane correlation distances related to the patterns observed were calculated from the *Q*_y_ spacing of the peaks in each sample. Signals were integrated across the horizontal *Q*_y_ axis and over a *Q*_z_ range corresponding to the angle of specular reflection, adjusted to include a single row of peaks in each integration box (Figure 2D, integrated regions shown by the yellow boxes in Figure 2C). The linear plot of the *Q*_y_ position of the maxima against the order of the peaks shows that the maxima are equidistant in *Q*_y_, and the slope of the straight lines fitted through the data points (the *m* factor in the line equation *y = mx + c*) corresponds to the average Δ*Q*_y_ (Figure 2E). The slopes obtained from the different samples yielded correlation distance values of 2124, 1081, and 609 Å for the three particles analyzed, in close agreement with the monolayer thickness values extracted from NR and the values obtained by DLS, indicating that the correlation distances obtained from the model-independent analysis of the GISAXS scattering patterns contain information on the form factor of the particles.

Using the information on particle size obtained from NR measurements, simulated GISAXS signals were generated using the BornAgain software^33^ that implements the distorted wave Born approximation framework to calculate the scattering signal from an ensemble of particles on a surface (Figure S4). The average lattice distances, the distance between the centers of the spheres, used in the simulations were 620, 1100, and 2190 Å for the three sizes, and lower densities of spheres were assumed as the size of the particles decreased (Table S2), following the trend observed in the NR data. The simulated scattering was overall in good agreement with the data, although providing only qualitative information on the average lattice distances and particle densities as the values displayed were not extracted from a fitting routine as in the case of the NR data.

### NanoSLB Formation by QCMD and Fluorescence Microscopy

**Figure 3:**
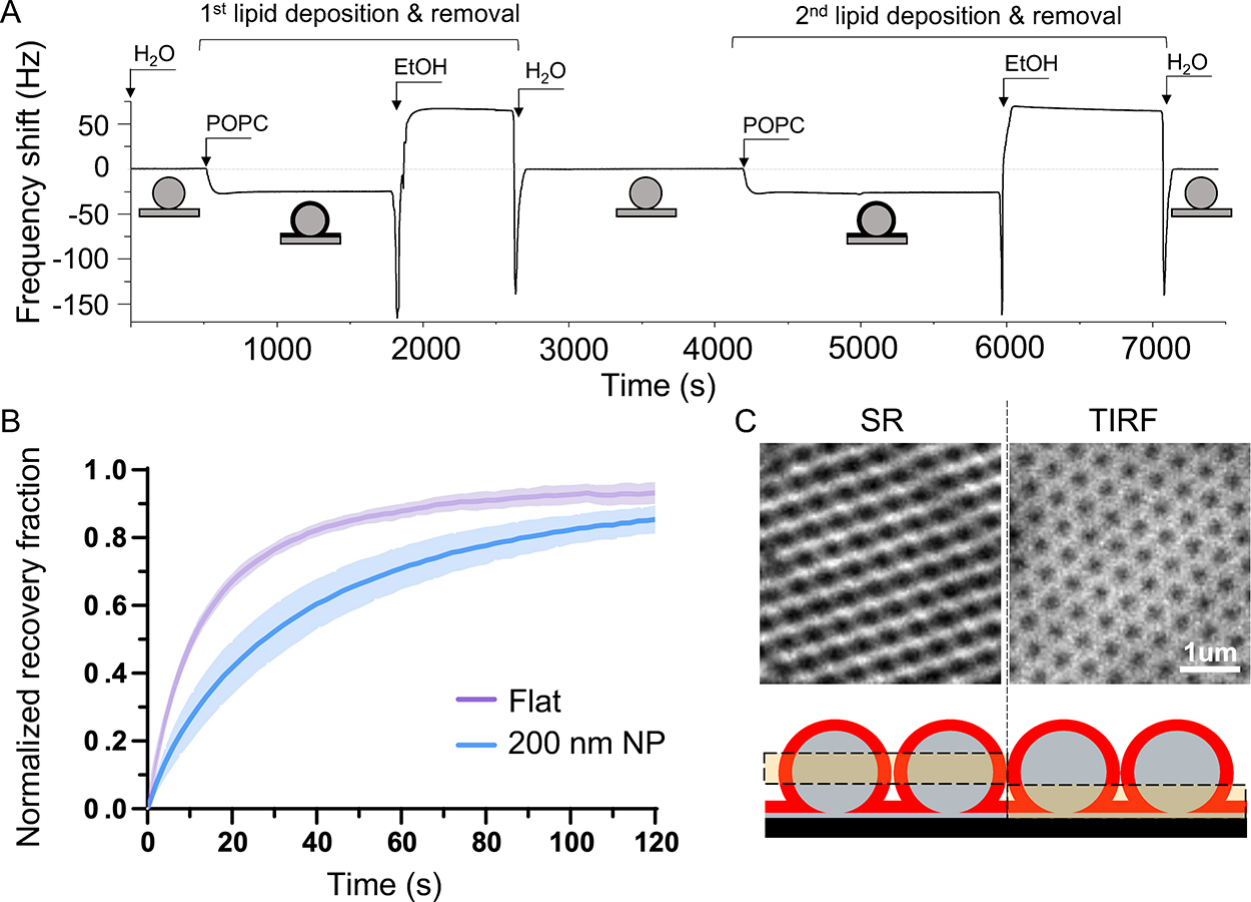
Formation of nanoSLB measured by QCMD and fluorescence microscopy. (A) QCMD trace monitoring the deposition of POPC onto a silica sensor coated with a monolayer of NP. Labels indicate the injection of different solutions in the QCMD flow module. The light gray flat dotted line is a guide to the eye set at zero Hz frequency shift, showing that lipids can be deposited and removed without affecting the NP layer. (B) Recovery of the fluorescence intensity after photobleaching showing the average of different measurements from five different regions of interest with the standard deviation represented by the shaded area. (C) Super-resolution (SR) microscopy and total internal reflection fluorescence (TIRF) images of NP array coated with a POPC bilayer; the highlighted boxes in the cartoon show the regions where each fluorescent microscopy technique is most sensitive. In SR and TIRF imaging, images were acquired on nanoSLB formed on particles with a larger nominal diameter (400 nm) that provides the optimal resolutions for the SLBs at different Z-positions in the focal plane.

The monolayers containing the largest NP displayed the highest particle density and were therefore chosen as the substrate for the study of lipid bilayer deposition. Lipid deposition via vesicle fusion was monitored by QCMD, which was used to compare the process of bilayer formation on conventional “flat” silicon oxide sensors and on sensors coated with the 200 nm NP monolayer (Figure 3A). The frequency shift (Δ*f*) observed after injecting POPC vesicle and rinsing with water was −24.2 ± 0.7 and −25.5 ± 0.8 Hz on the flat and NP-coated sensors respectively, in line with the values reported previously for the formation of lipid bilayers on flat silicon oxide sensors as well as on QCMD sensors coated with NP.^18^ The shift in dissipation (Δ*D*) amounted to +0.4 ± 0.3 ppm on the flat and −5.8 ± 1.2 ppm on the NP-coated surfaces (Figure S5A). The slightly higher Δ*f* value recorded on the NP sample shows a marginally higher amount of lipids adsorbed while the negative Δ*D* indicates that lipid addition affects the properties of the NP layer, suggesting that intercalation of lipids in between the NP increases the overall stiffness of the nanoSLB layer.^34^ The surfaces were then rinsed with ethanol to remove the POPC, which in both cases caused Δ*f* and Δ*D* to return to the baseline measured at the beginning of the experiment, indicating complete removal of the lipids by the ethanol wash, with the NP array remaining unperturbed. A second cycle of lipid deposition and removal was carried out yielding Δ*f* and Δ*D* values in line with the shifts observed in the first cycle, showing full reusability of the NP array as a substrate for the formation of nanoSLBs (Figure 3A). The diffusivity of the POPC molecules within the nanoSLB was investigated using fluorescence recovery after photobleaching (FRAP). Compared to SLB formed onto flat glass surfaces, the measured lipid diffusion after bleaching was slower on the bilayers assembled on the NP-coated substrate (Figure 3B). The final recovery was nonetheless comparable between the two substrates after 120 s from the bleaching (Figure S5B), indicating that lipid mobility in the nanoSLB, although more restricted, was still largely retained both in the planar bilayer and around the NP. Further experiments are required to understand to what extent diffusion takes place directly between neighboring NPs compared to diffusion mediated by the planar underlying SLB. Imaging of the nanoSLB by super-resolution (SR) and total internal reflection fluorescence (TIRF) microscopy provided a clear picture of the fluorescent bilayer and the NP array. The intense fluorescence in the TIRF signal suggests the presence of a lipid bilayer coating the area in between the particles on the flat substrate while the fluorescence signal in SR, which comes from higher up in the sample, indicates the presence of a ring of lipids around the particles (Figure 3C).

### Structure of NanoSLB by Specular NR

**Figure 4:**
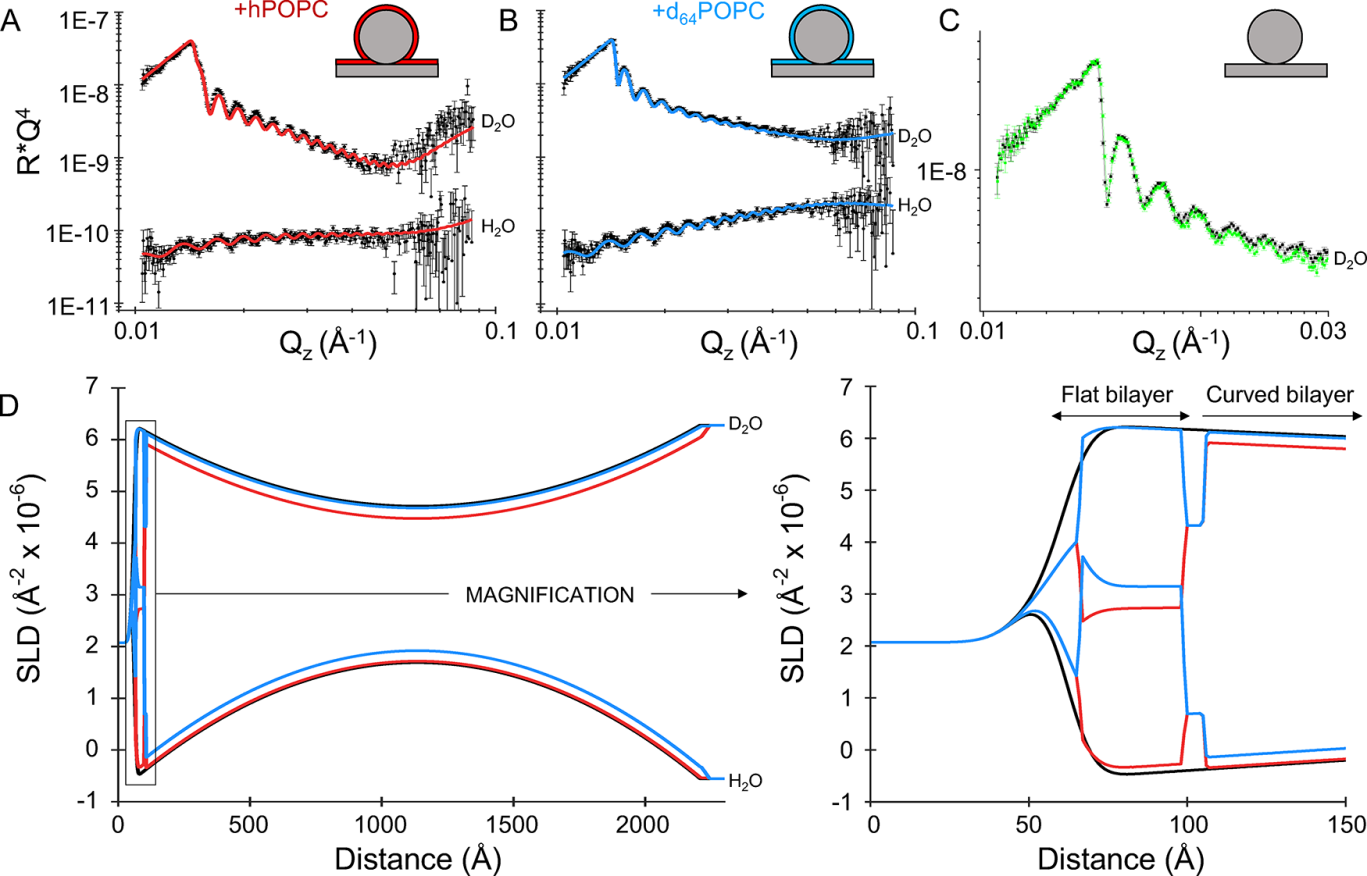
Vertical structure of the lipid-coated NP array. (A) NR data (points) and best fit (red lines) of nanoSLB formed with hPOPC in H_2_O and D_2_O. (B) NR data (points) and best fit (blue lines) of the nanoSLB formed with d_64_POPC in H_2_O and D_2_O. (C) NR data from NP monolayer in D_2_O before (black) and after (green) one cycle of lipid deposition and removal, showing recovery of the reflectometry signal upon EtOH rinsing. (D) SLD profiles describing the NP monolayer in the absence of lipids (black dashed lines) in the presence of hPOPC (red lines) and in the presence of d_64_POPC (blue lines) obtained from the constrained fits with six datasets, parameters of the fits are shown in Table S3. The expanded region shows a magnification of the flat SiO_2_ interface highlighting the formation of the planar SLB on the flat silicon substrate.

To access information on the structure of the lipid bilayer formed on the NP array, the nanoSLB was characterized by NR. To fully exploit the ability of neutrons to differentiate between hydrogenous and deuterated molecules, the process investigated by QCMD (Figure 3A) was replicated on the neutron beam line using first hydrogenous hPOPC, followed by regeneration of the surface with ethanol and deposition of tail deuterated d_64_POPC. After each lipid assembly, the sample was characterized both in H_2_O and in D_2_O. The reflectivity datasets collected in H_2_O and D_2_O on the bare NP array, the hydrogenous nanoSLB, and the deuterated nanoSLB were then fitted to a common model of the interface where the structural parameters describing the SiO_2_ NP monolayer were shared across the six conditions, while the SLD of the lipids and their volume fractions were allowed to vary (see Figure 2D,E for the fits and SLD of the NP monolayer before lipids addition). The model that produced the most accurate fit to the reflectivity data from the nanoSLB samples included a lipid bilayer coating the entire nanoparticle surface as well as part of the flat surface of the silicon substrate supporting the particles, as suggested by the fluorescence microscopy data (see the Supporting Information and Figure S1 for a detailed description of the model used). Addition of hPOPC vesicles to the NP array caused a prominent shift of the reflectivity profile measured in D_2_O while leaving the signal recorded in H_2_O mostly unaffected, as expected from the deposition of hydrogenous material at the interface (Figure S6A). Conversely, addition of d_64_POPC resulted in a large shift in the reflectivity measured in H_2_O but no significant changes in D_2_O (Figure S6B). Similarly to that observed with QCMD, rinsing the nanoSLB with ethanol between bilayer depositions reverted the reflectivity back to the initial profile measured before lipid addition, confirming nearly complete removal of lipids while preserving the intact NP monolayer structure (Figure 4C).

Both the hydrogenous and the deuterated systems were described using the same set of shared parameters for thickness and hydration, with the only difference being the SLD of the lipid tails. The parameters obtained from the fits to the NR data described the formation of a lipid bilayer adhering to the NP, which coated the entire surface of the spheres with a high coverage as indicated by the low volume fraction of water detected in the tail region, below 1% (Table S3 and Figure S7). Additionally, a planar lipid bilayer formed on the silicon crystal surface underlying the NP monolayer, with an overall coverage of ∼55%, indicating the formation of a less complete lipid layer in comparison with the bilayer coating the NP. On planar SiO_2_ surfaces, vesicles tend to fuse into high-coverage SLBs once a critical surface density of intact vesicles is reached,^35−37^ whereas this accumulation process is not required on the surface of SiO_2_ NP, where vesicles have been suggested to fuse one by one as indicated by cryo-electron microscopy images.^38^ The lower coverage of the underlying planar SLB is likely to result from the hindrance of the large NP which interfered with the accumulation of vesicles that leads to the formation of homogeneous and continuous lipid bilayers, resulting in a suboptimal coverage. Along with the differences in coverage, the structural data revealed an overall ∼9 Å thinner nanoSLB (∼37 Å) compared to the planar bilayer (∼46 Å). Although both tails and headgroup thickness parameters obtained from the error analysis displayed an overlap of the confidence interval regions as displayed by their posterior distributions (Figure S7), a comparable difference was recently also reported on a similar system studied by NR by Armanious et al.^39^ While the parameters obtained for the planar POPC SLB are in excellent agreement with previously reported values,^40,41^ the values obtained for the thickness of the nanoSLB seem to indicate a suboptimal packing of the lipids around the spheres that could have resulted from the curvature imparted by the NP substrate. Results on bilayer thickness estimates obtained from SANS on POPC vesicles of varying size in bulk do not show significant thickness differences between vesicles in this size range.^42^ Therefore, the effect observed on the structure of the nanoSLB is likely to be a consequence of the interaction with the NPs and their surface chemistry, possibly due to differences in the overall density of silanol groups or differences in roughness between the planar SiO_2_ substrate and the NP SiO_2_ interface. Additionally, it has to be noted that the thickness of the nanoSLB is not directly measured by NR but is extracted from the volume fraction contributions of the nanoSLB to the SLD of the slices used to model the interface as described in the Supporting Information. The NR data yielded comparable results in terms of differences in bilayer thickness and coverage parameters when fitted with a model of the nanoSLB described as a single homogeneous layer (Figure S8 and Table S4). Although the contributions of the headgroups to the overall reflectivity are small, by parametrizing the nanoSLB with the same layout as that used for the flat bilayer (i.e., with separate headgroups and tail regions as shown in Figures S1 and S7, as opposed to the single layer of Figure S8), the respective hydrophilic and hydrophobic regions of the planar and nanoSLB can share a common SLD value imposing an additional constraint on the model. Furthermore, treating the nanoSLB as a single layer does not reduce the number of parameters required to model the data in this case (Tables S3 and S4) and the posterior distributions of the bilayer parameters obtained display a more symmetric distribution when the nanoSLB is parametrized as a conventional lipid bilayer with distinct regions.

### Additional Characterization by Off-Specular Neutron Scattering

**Figure 5:**
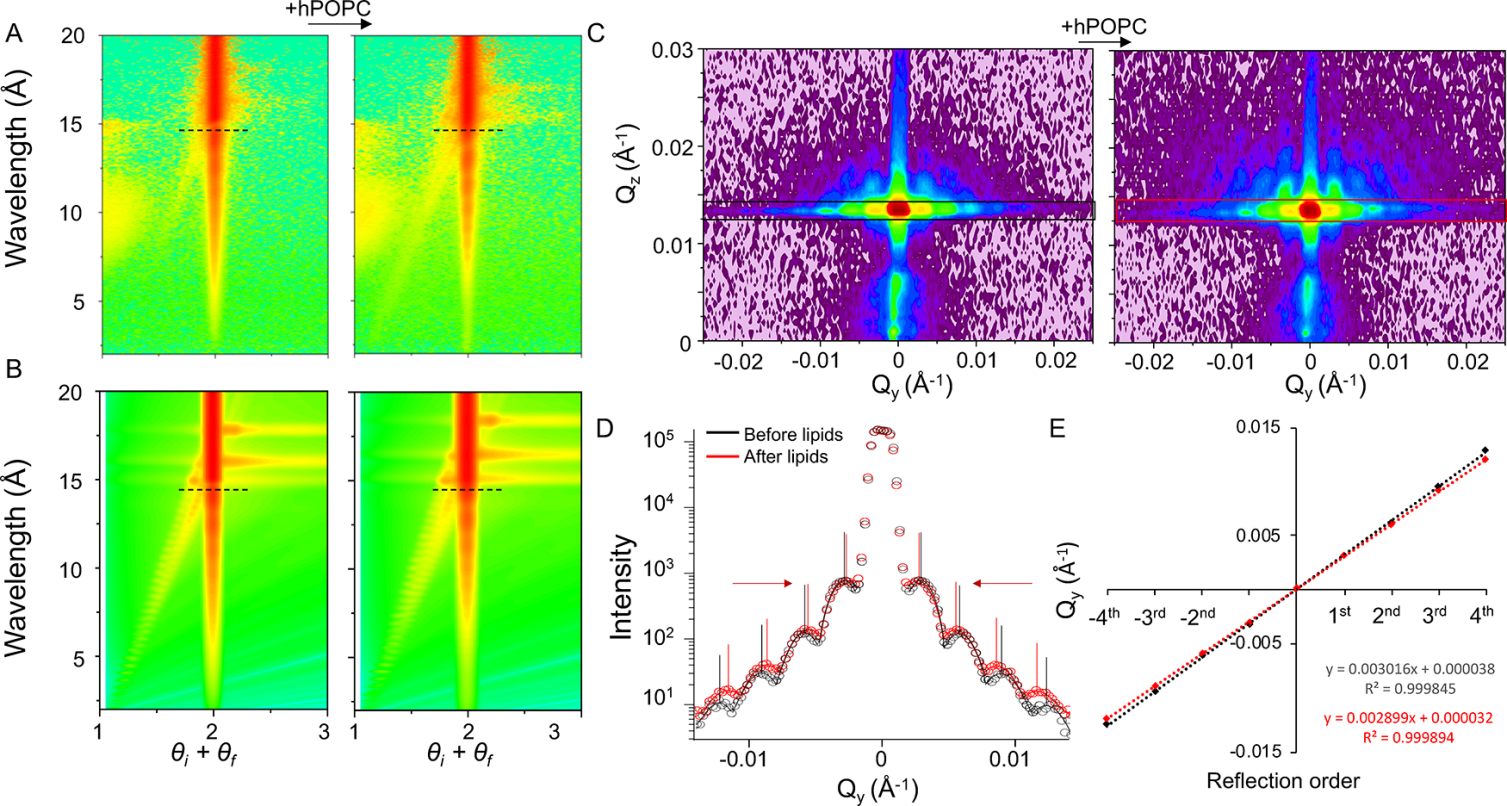
Effect of NanoSLB formation on GISANS and off-specular NR signals. (A) Off-specular NR data in D_2_O before (left) and after (right) the addition of hPOPC to the NP array (dashed lines indicate the position of the critical edge). (B) Simulations of off-specular NR in the absence (left) and presence (right) of lipids coating the NP; θ_*i*_ and θ_*f*_ are the incident and reflected angles, respectively. (C) GISANS detector images of NP monolayer in D_2_O before (left) and after (right) the addition of hPOPC. (D) Integration across *Q*_y_ of the GISANS images corresponding to the colored boxes in (C) before (black) and after (red) addition of lipids. Black and red vertical lines indicate the maxima position of individual gaussian curves fitted to the peaks, and red arrows indicate the direction of these peaks shift upon lipid addition. (E) Linear fits to the maxima positions along *Q*_y_ of the peaks before (black) and after (red) formation of hPOPC nanoSLB shown in (D). The equations of the fits and the *R*^2^ values are displayed in the graph. The *x* coefficient in the equations corresponds to the average separation of the peaks, Δ*Q*_y_.

The NR curves measured in D_2_O showed a strong off-specular signal captured in the 2D detector images as horizontal stripes in the region of total reflection (Figure 5A), which caused the intensity dips visible in the specular reflectivity signal below the critical edge (Figure S6, inset). This is similar to that observed in the case of resonators, characterized by a potential well, formed of a region of low SLD in between two regions of high SLD.^43,44^ In the case of the spherical NP system under investigation here, the increasing volume fraction of silicon oxide (SLD of SiO_2_ = 3.47 × 10^–6^ Å^–2^) toward the center of the NP monolayer generates a region of low SLD in comparison to the D_2_O-rich regions above and below the monolayer center (SLD of D_2_O = 6.35 × 10^–6^ Å^–2^), which explains the appearance of the resonances observed. The addition of hPOPC caused both an increase in the intensity of the off-specular scattering and a shift in the *Q*_z_ position of the resonances (Figure 5A). Simulations of the off-specular data^45^ reproduced qualitatively the signal change observed for the NP monolayer before and after addition of lipids (Figure 5B) confirming the validity of the specular reflectivity model used to fit the data. The off-specular scattering simulations complemented the GISAXS results indicating higher packing densities in the larger NP arrays. To reproduce the measured off-specular intensities of the 100 and 50 nm particles (Figure S8), micrometer-sized D_2_O clusters had to be included in between the NPs in the calculations, while these large D_2_O clusters were not required to simulate the signal from the 200 nm particles, indicating the absence of large defects in the monolayer made with the larger particles.

While off-specular reflectometry is sensitive to changes in the SLD distribution over micrometer length scales, GISANS measurements are sensitive to variations of the in-plane SLD distribution over nanometer length scales.^46^ Following up on the GISAXS data collected on the dry NP arrays, the process of nanoSLB formation was investigated by GISANS, which enables measurements of grazing incidence small-angle scattering on buried (e.g., wet) biological thin films. The analysis of the integrated peak positions yielded a correlation distance of 2083 Å prior to the addition of lipids, in line with the values obtained from DLS, GISAXS, and NR (Figure 2). A comparison of the GISANS signals before and after the addition of hPOPC to the NP array in D_2_O revealed an increase in the intensity of the overall scattering in the 2D detector image, in line with the increased contrast in the sample caused by the addition of hydrogenous lipids in D_2_O (Figure 5C,D). Notably, along with the change in intensity, the adsorbed lipids caused a shift of the maxima positions in *Q*_y_, with a reduction of the average inter-peak separation, resulting in an increase in the relative correlation distance from 2083 to 2167 Å, corresponding to an overall increase of 84 Å in the particle diameter, as calculated from the linear regression of the peak positions (Figure 5E). According to the structural information obtained from the NR data, the total bilayer thickness formed around the NP amounts to ∼37 Å; thus, the expected increase in the apparent NP diameter upon bilayer formation would be ∼74 Å, close to the 84 Å obtained from the model-free GISANS analysis, which corroborated the NR results.

## Conclusions

The properties of nanoSLBs have been investigated both in bulk^47−51^ and at interfaces^18,20,24,52^ with a wide range of biophysical techniques. Here, we provide a close-up on the molecular structure of coexisting flat and curved lipid bilayers assembled on nanoparticle arrays, using a combination of surface-sensitive imaging and scattering techniques. Together, the data demonstrate the possibility of using large NP arrays assembled via an accessible bottom-up method that does not involve complex nanofabrication, as a substrate for the formation of high-coverage curved lipid bilayers over large surfaces. The combination of scattering and imaging methods granted access to accurate structural information on the vertical and in-plane structure of the nanoSLB, revealing the coexistence of planar and curved regions that displayed different lipid packing between the two arrangements. The nanoSLB platform described here provides a new tool for the study of fundamental and applied aspects of curvature-dependent membrane phenomena, opening new possibilities to investigate curvature-induced lipid phase separation and protein segregation using both grazing incidence neutron scattering coupled with isotopic labeling, as well as more widespread fluorescence-based imaging techniques.

## Supporting Information Available

Materials and methods with explanatory figures; description of NP arrays preparation; description of experimental setup for AMF; fluorescence microscopy; DLS, GISAXS, GISANS, and NR; detailed description of NR data modeling; DLS results; Bayesian error analysis of reflectometry fits and corresponding tabulated parameters; GISAXS simulations; additional details on QCMD and fluorescence microscopy measurements; and additional off-specular scattering simulations (PDF)

## Abbreviations

NP: nanoparticles
SLB: supported lipid bilayer
nanoSLB: nanoparticle-supported lipid bilayer
SLD: scattering length density
NR: neutron reflectometry
GISAXS: grazing incidence small-angle X-ray scattering
GISANS: grazing incidence small-angle neutron scattering
FRAP: fluorescence recovery after photobleaching
QCMD: quartz crystal microbalance with dissipation
AFM: atomic force microscopy
TIRF: total internal reflection fluorescence
DLS: dynamic light scattering
