# Supplementary material for: Structural Characterization of Nanoparticle-Supported Lipid Bilayer Arrays by Grazing Incidence X-ray and Neutron Scattering
Source: ACS Appl Mater Interfaces. 2023 Jan 10;15(3):3772–80. doi: 10.1021/acsami.2c18956 (PMC9880997; doi:10.1021/acsami.2c18956)
Supplement: Supplementary file 1 — am2c18956_si_001.pdf [file am2c18956_si_001.pdf]

Supplementary Information, Materials and Methods for:

Structural Characterisation of Nanoparticle-Supported Lipid Bilayer Arrays by Grazing Incidence X-ray and Neutron Scattering

Nicolò Paracini[1*], Philipp Gutfreund[2], Rebecca Welbourn[3], Juan Francisco Gonzalez-Martinez[1], Kexin Zhu[4], Yansong Miao[4], Nageshwar Yepuri[5], Tamim A Darwish[5], Christopher Garvey[6], Sarah Waldie[1], Johan Larsson[1], Max Wolff[6], and Marité Cárdenas[1,4*]

[1] Department for Biomedical Science and Biofilms – Research Center for Biointerfaces, Malmö University, Faculty of Health and Society, 205 06, Malmö, Sweden
[2] Institut Laue-Langevin (ILL), 38000 Grenoble, France
[3] ISIS Neutron & Muon Source, STFC, Rutherford Appleton Laboratory, Harwell, Oxfordshire, OX11 0QX, UK.
[4] School of Biological Sciences, Nanyang Technological University, Singapore.
[5] National Deuteration Facility, Australian Nuclear Science and Technology Organization (ANSTO), Australia
[6] Heinz Maier-Leibnitz Zentrum (MLZ), Technische Universität München, Lichtenbergstraβe 1, 85748 Garching, Germany
[7] Department of Physics and Astronomy, Uppsala University, Box 516, 751 20, Uppsala, Sweden

*Email: nicolo.paracini@mau.se, marite.cardenas@mau.se

Materials:

1-palmitoyl-2-oleoyl-sn-glycero-3-phosphocholine (POPC) received from Larodan (Sweden), tail deuterated (d_{64}) POPC was produced by the National Deuteration Facility of the Australian Nuclear Science and Technology Organisation (ANSTO, Australia) and purified as previously described [1]. Non-porous silica nanoparticles (NP) of 50 nm, 100 nm, 200 nm and 400 nm nominal diameter suspended in milliQ water were sourced from Alpha Nanotech (Canada). Deuterium oxide (D_2O) and cetyltrimethylammonium bromide (CTAB) were from Sigma Aldrich (Merk, Germany). The fluorescent lipid dye 1,2-dioleoyl-sn-glycero-3-phosphoethanolamine-N-(lissamine rhodamine B sulfonyl) (18:1 Liss Rhod PE) was from Sigma Aldrich (Merk, Germany). Silicon crystals (100) 60x80x10 mm, polished to a rms roughness <5 Å were from Sil'tronix (France). Glass slides for fluorescence microscopy with a diameter of 25 mm and a thickness of 0.16-0.19 mm were from Paul Marienfeld GmbH & Co (Germany) and were enclosed in Attofluor cell chamber from Invitrogen (Thermo Fisher Scientific inc. USA). All chemicals were used without further purification.

Methods:

Langmuir-Schaefer assembly of NP arrays:

NP preparation: Silica NP were first exchanged from milliQ water into ethanol by centrifugation (10.000 rpm for 15 min) and resuspension (vortexing and sonication) until suspended in >99% ethanol according to manufacturer indications. After extensive vortexing and sonication in a cold bath, a 10 mg/ml suspension of particles was mixed 1:1 with a 2 mM ethanolic solution of CTAB to obtain a final concentration of 5 mg/ml NP and 1 mM CTAB in ethanol.

Trough set-up: A substrate (silicon crystal for scattering experiments, QCMD sensor, or glass slide fluorescent microscopy experiments) was cleaned by sonication in 2% SDS (10 min)

followed by rinsing under milliQ water and sonication in hot ethanol (20 min), dried under N_2 and placed in a UV/ozone cleaner for 20 minutes. The substrate was then rinsed thoroughly under milliQ water, dried with N_2 and placed face-up on a holder inside a thoroughly cleaned custom-built Teflon Langmuir trough placed inside a fumehood (total trough surface 380x100 mm). The trough was filled with milliQ water until the water completely submerged the substrate's surface. The water surface was cleaned by aspiration with a nozzle connected to a pump. The water level was then adjusted by adding or removing water from behind the Teflon barrier so that the water level was only a few millimetres above the substrate surface.

NP isotherms and deposition: Prior to spreading the NP onto the water surface, the 5 mg/ml NP, 1 mM CTAB ethanolic suspension was sonicated in a cold bath for 30 min. For the different NP sizes, volumes to deposit were calculate by estimating the number of NP required to yield a monolayer with an area corresponding to ~50% of the trough surface. The suspension was then deposited in a drop-wise manner onto the water surface using a 200 uL pipette from a height < 1 cm. The monolayer was left equilibrating for 15 min and then compressed to the target surface pressure (between 5-10 mN/m for all monolayers). Once the surface pressure stabilised, the compression was stopped, and the water level was lowered by slowly removing the subphase with a serological pipette connected to a pump from behind the barrier to avoid perturbing the monolayer until the substrate was completely emerged. The substrates were then left untouched inside the fume hood until dry.

Atomic Force Microscopy: A commercial Atomic Force Microscopy setup (MultiMode 8 SPM with a NanoScope V control unit, Bruker AXS, Santa Barbara CA) was used for imaging the transferred NP monolayer on the silicon wafer. Images were acquired by operating the AFM using PeakForce Tapping mode. For imaging in the PeakForce Tapping mode, cantilevers with a nominal resonance frequency between 320 and 364 kHz were used (RTESP7, Veeco Probes, Camarillo, CA).

Dynamic Light Scattering: Dynamic light scattering (DLS) measurements were performed on a Zetasizer Ultra (Malvern Panalytical, United Kingdom) equipped with a 633 nm red laser. Size measurements were performed in backscattering geometry with the detector positioned at 173 degrees relative from the source. NP samples were diluted to 0.2 mg/ml, sonicated for 30 minutes in a Branson 5800 bath sonicator (Branson Ultrasonics, Slovakia) and measured in DTS0012 12 mm polystyrene cuvettes in a total volume of 1 ml. Size and polydispersity index were obtained from the measurements using the Zetasizer Ultra-Pro ZS Xplorer v1.31 integrated analysis software provided with the instrument. Measurements were performed in triplicates for each particle size and are displayed here as average and standard deviation.

Fluorescence Microscopy: For super-resolution microscopy, images were acquired on a spinning disk system (Gataca Systems) based on a Nikon Ti2-E inverted microscope which also equipped with a super resolution module (Live-SR; Gataca systems) based on structured illumination with optical reassignment technique and online processing. The microscope was equipped with a sCMOS camera (Orca-Fusion; Hamamatsu), a confocal spinning head (W1; Yokogawa), a 100x 1.45 NA Plan-Apo objective lens. TIRF microscopy and FRAP images were acquired on the same microscope using iLAS ring-TIRF or FRAP system (GATACA Systems). All images were acquired using MetaMorph software (Molecular Devices, LLC, Sunnyvale, CA). SR and TIRF images were acquired on nanoSLB formed on particles with a larger nominal diameter (400 nm) that provides the optimal resolutions for the SLBs at different Z-positions, including the nanoSLB and the planar SLB on the underlying glass surface.

QCMD measurements: QCMD measurements were performed on a QSense Analyzer (Biolin Scientific, Sweden). Of the 4 flow modules two were equipped with flat silicon oxide sensors and two with sensors coated with a 200 nm NP array and were left equilibrating in water until the frequency and dissipation signals stabilised. Solutions were flushed through the cells using a peristaltic pump at a speed of 0.1 ml/min. Frequency and dissipation shifts of the 7th harmonic were measured for the lipid deposition and removal processes. Values reported in the text are the average and standard deviation of 4 lipid depositions on each sensor.

GISAXS measurements: GISAXS measurements were performed on a XEUSS 3.0 (Xenocs, France) equipped with a CuKα source (wavelength 1.54 Å) and a Pilatus 300K detector (Dectris, Switzerland) placed at 1700 mm from the sample. The beam was collimated in ultra-high resolution mode using slits of 300 x 150 μm and the angle of incidence was 0.2°. Samples assembled on silicon crystals, were measured for 3 hours in ambient conditions.

GISAXS and GISANS peak analysis: The 2-dimensional GISAXS and GISANS detector images were integrated across the horizontal Q_y axis and over a Q_z range corresponding to the angle of specular reflection. The Q_z range was adjusted to include a single row of peaks in each integration box and each peak obtained from the integration was fitted to a single Gaussian in order to obtain the Q_y positions of the maxima. The Q_y values of the peak maxima were plotted against the ordinal number of the peaks and for each data set a straig ht line was fitted through the points to obtain the slope of the line from the line equation $y = mx + c$. The m coefficient corresponds to the average ΔQ_y, and the values obtained were used to calculate the in-plane correlation distances in the samples by using the relation $d = 2\pi/\Delta Q_y$

GISAXS simulations: GISAXS simulations were performed using the BornAgain software [2]. A virtual instrument was created in the software that replicated the specifics used for data collection and described above as well as a background of Poisson noise. A model was set up reproducing a layer of silicon oxide spheres (X-ray SLD 1.88 e-5 $Å^{-2}$) in a finite 2D hexagonal lattice on a 10 Å thick silicon oxide layer supported by an infinitely thick silicon substrate (X-ray SLD 2.00 e-5 $Å^{-2}$). The finite 2D lattice was defined by a radius parameter and the distance between the spheres given by a lattice parameter. The unit cell of the finite lattices were averaged over the lattice rotation angle χ, i.e. averaging the signal for all possible in plane orientations of the hexagonal unit cells. BornAgain calculated the analytical solution of the interference of the X-ray radiation under the distorted wave Born approximation. Values of the simulations were changed manually until reasonable agreement with the data was found and therefore do not represent fits but rather qualitative estimates. Parameters input for the simulations are given in **Table S2**.

Neutron Reflectometry and GISANS:

Sample preparation: Silicon crystals coated with the NP arrays were placed in the UV-ozone cleaner for 5 min, rinsed with milliQ water and dried under N_2, prior to assembly into solid liquid cells. POPC vesicles were prepared by thin film hydration and sonication in milliQ water at a concentration of 0.2 mg/ml. Immediately prior to vesicles injection in the cells the POPC suspension was diluted 1:1 with a 4 mM $CaCl_2$ solution to yield a final concentration of POPC 0.1 mg/ml and 2 mM $CaCl_2$. Vesicles were injected using a syringe pump at a flow rate of 1 ml/min. The injected vesicles were collected from the solid-liquid cell outlet and injected again in the opposite direction to maximise the lipid deposition.

NR Measurements: Neutron reflectometry measurements were carried out on the Figaro beamline at the Institute Laue Langevin (Grenoble)[3]. Measurements were performed using a white neutron beam with wavelengths between 2 and 20 Å at two angles of incidence for the NP arrays (1° and 3.2°). For the nanoSLB s only the 1° angle was measured due to time constrains. Specular and off-specular signals were recorded on the area detector. H_2O and D_2O were flushed through the cells at 1 ml/min for 900 s to exchange solutions and EtOH washes were performed for 1200 s at the same flow rate.

Specular NR data analysis: Neutron reflectometry curves were fitted with the Rascal software using the 2019 version (https://sourceforge.net/projects/rscl/). The reflectometry datasets were fitted to a mathematical model of the interface describing a silicon substrate covered with a layer of silicon oxide on top of which a monolayer of spheres was built from stacked thin slices, each one characterised by a thickness, SLD and roughness parameter as further described below and in **Figure S1**. For the 50 nm and 100 nm NP arrays, two datasets per sample (collected in H_2O and in D_2O) were constrained to fit to a common structure of the monolayer, only allowing the SLD of the solvent to vary between the parameters of the different contrasts. For the 200 nm NP used to assemble the nanoSLB, the model was expanded to fit together six datasets which all shared the same structure of the spheres (i.e. thickness and spheres volume fraction). Two datasets were the NP array in H_2O and D_2O, two the nanoSLB assembled using hPOPC and two the nanoSLB assembled using tail deuterated d_{64}POPC. datasets were fitted to the model using the Nelder-Mead algorithm available on RasCAL which minimises the chi^2 function describing the difference between the data and the calculated reflectivity from parameters that were allowed to vary between the ranges described in **Table S1, S3** and **S4**. Confidence intervals on the parameters were obtained using the Markov chain Monte Carlo (MCMC) methods implemented within RasCAL once the chi^2 could not be further minimised. The distribution of priors was assumed to be flat for all parameters and the posterior distribution was obtained from 10000 iterations and 1000 burn-in points. 65% confidence intervals, approximating one standard deviation, were calculated from three independent repeat runs.

Models used to fit the NR data: NR data was fitted to a slab model of the interface built using the custom layer option in Rascal. The model described the interface between two infinite slabs representing the silicon substrate (SLD 2.07×10^{-6} Å^{-2}) and the solution composed either of H_2O (expected SLD -0.56×10^{-6}Å^{-2}) or D_2O (expected SLD -6.35×10^{-6}Å^{-2}). The interface between the solution and silicon layer was composed of a slab adjacent to the silicon modelling the native SiO_2 layer (SLD 3.47×10^{-6}Å^{-2}) on top of which an array of spheres was placed, each sphere enclosed in a hexagonal prism with the base aligned to the SiO_2 interface, its sides equal to $d/\sqrt{3}$ and a vertical height equal to d, where d is the particles diameter (**Figure S1A**) The box was sliced into slabs of constant thickness, with slabs thicknesses varying depending on the size of the particles: 100, 200 and 1000 slices were used for the 50 nm, 100 nm and 200 nm respectively. In each slab within the box, the volume fraction occupied by the sphere was calculated as a function of the distance from the interface along the axis normal to the substrate and the remaining volume fraction within the prism was filled with the aqueous solvent. This model accounts for hexagonally packed spheres enclosed in a regular hexagon with side $d/\sqrt{3}$ and a 'sphere coverage' parameter defined the total volume occupied by the prisms enclosing the spheres whilst the remaining volume fraction in between prisms was filled with solvent.

To fit the data from the lipid coated system, the sphere model was expanded to fit together the six datasets obtained from the 200 nm bare particles, the hydrogenous nanoSLB and the

deuterated nanoSLB, each sample characterised in both H_2O and D_2O. The model accounted for the contribution of the lipid bilayer coating the spheres and a planar lipid bilayer adsorbed on the underlying SiO_2 substrate. To do so, the lipid-coated sphere was divided either into 7 different regions as described in **Figure S1** to model the nanoSLB using the tripartite head-tails-head layout used for the planar bilayer, or into 5 regions to model the nanoSLB as a single homogeneous layer as shown in **Figure S8**. The SLD of each section was calculated by adding the contributions of each component in each slice obtained by multiplying its volume fraction (Ø) by its SLD. In turn the SLD of the lipid bilayer components (lipid tails and lipid headgroups) were defined by the calculated values for POPC tails (SLD -0.3 × 10^{-6}Å^{-2}) and POPC headgroups (SLD 1.98 × 10^{-6}Å^{-2})[4]. A hydration parameter associated with each region of the bilayer accounted for the contribution of the volume fraction of solvent to the SLD of the tails and headgroup regions of the bilayer. The six contrasts were constrained to fit using the shared volume fraction and size parameters of the spheres which constrained the nanoSLB volume fractions to the surface coverage of the SiO_2 nanoparticles. The model was further constrained to satisfy the assumption that the hydration of the headgroup region can only be higher than the hydration of the tail region given the molecular constraints. This was accounted for in the model by using the hydration of the tails region as the minimum volume fraction of solvent in the corresponding headgroups and implementing an additional hydration parameter for the headgroups defined as 'additional headgroup hydration'. The total volume fraction of solvent present in the headgroups was therefore given by the sum of the hydration of the tails and the additional hydration of the headgroups. Finally, the hydrogenous and deuterated bilayers shared the same hydration parameters for the respective headgroup and tail regions in the nanoSLB and the planar bilayer.

To generate the model of the interface formed by the NP and the lipid coating as shown in **Figure S1**, we considered the volume contribution of the various components in slices away from the surface. The contribution of the NP sphere and the lipid coating give a thin cylinder corresponding to the NP and three hollow cylinders corresponding to the inner and outer head groups regions and the tail region, with the radii of these (x_1, x_2, x_3 and x_4) dependent on the distance from the surface.
The model was parametrized using the following mathematical description:

Given:
t = thickness of each slice
i = slice index
R_1 = radius of the sphere

for slices up to the midpoint of the NP (i.e. if $t \times i < R_1$) then:
$b = R_1 - t \times (i - 0.5)$

for slices above the midpoint of the NP (i.e. if $t \times i > R_1$) then:
$b = -(R_1 - t \times (i - 0.5))$

therefore, across the whole diagram:
$b^2 = (R_1 - t \times (i - 0.5))^2$

and by Pythagoras:
$x_1^2 = R_1^2 - b^2$
$x_2^2 = R_2^2 - b^2$

$x_3^2 = R_3^2 - b^2$
$x_4^2 = R_4^2 - b^2$

where:
$R_2 = R_1$ + inner headgroup thickness
$R_3 = R_2$ + tails thickness
$R_4 = R_3$ + outer headgroup thickness

For fitting the data of the 50 and 100 nm bare particles the hexagonal prism containing the sphere was sliced in 100 and 200 slices respectively so that each slice had a thickness of ~5 Å with an interfacial roughness fixed to 1 Å which represents a gaussian smearing of the interface between the slabs. For the 200 nm particles the spheres were sliced into 1000 layers to yield a thickness of ~2 Å per slice with a slice roughness set to 0.4 Å. The finer slicing of the 200 nm was necessary to provide enough resolution to model the SLD distribution of thin regions of the planar bilayers such as the tails and headgroups, the latter being only ~5 Å thin.

The slice roughness parameter and the number of slices were fixed as well as the SLDs of silicon, SiO_2, hydrogenous phospholipid tails and phospholipid headgroups [4]. Solutions SLDs were fitted to account for incomplete solution exchange in the solid liquid cells and were found to be within 2% of the theoretical values.

Off-specular NR analysis: Quantitative fits of the off-specular scattering patterns were performed using the algorithm described in [5]. The specular reflectivity model was coarse-grained to 7 layers in case of bare NPs and 9 layers in case of nanoSLB s of variable thickness and roughness reproducing closely the high-resolution SLD profile of the specular model. The top 6 slabs from this coarse-grained SLD profile, which correspond to the NP layer, are assumed to include cylindrical in-plane inhomogeneities with SLDs corresponding to SiO_2 (3.47 x 10^{-6}Å^{-2}) and D_2O (6.35 x 10^{-6}Å^{-2}), respectively, of variable radius corresponding to a sphere form factor. The positions of these cylinders were assumed to be out-of-plane correlated within these 6 slabs. The volume fraction of the respective cylinders are taken such that the mean SLD of the layer matches the SLD from the specular fits. Once the (protonated) lipid is added, two models gave equally good fits to the data starting from the fixed parameters from the bare NPs in D_2O: in the first case, the lipids are spread homogeneously between the NPs (as if they were in solution) and in the second a third phase (inhomogeneity) is present in between the SiO_2 cylinders and has a thickness and SLD of a hydrogenous lipid bilayer. Although both models yielded the same off-specular scattering patterns, only the latter model is physically relevant and in agreement with the results from the other techniques. The surface fraction of the defects used in the simulation of the off specular signal from 100 and 50 nm was in the 10% level if assuming micron-sized D_2O clusters. If assuming sub-micron clusters the surface fraction was up to 50%. From the off-specular signal it was not possible to distinguish between these two scenarios.

GISANS measurements: GISANS measurements were performed on D22 at the Institute Laue Langevin using a monochromatic beam with a wavelength of 6 Å. The sample was illuminated for 3 h at an angle of 0.35° and the area detector was placed 17600 mm away from the sample stage.

(1) Waldie, S.; Sebastiani, F.; Browning, K.; Maric, S.; Lind, T. K.; Yepuri, N.; Darwish, T. A.; Moulin, M.; Strohmeier, G.; Pichler, H.; Skoda, M. W. A.; Maestro, A.; Haertlein, M.; Forsyth, V. T.; Bengtsson, E.; Malmsten, M.; Cárdenas, M. Lipoprotein Ability to Exchange and Remove Lipids from Model Membranes as a Function of Fatty Acid Saturation and Presence of Cholesterol. *Biochim. Biophys. Acta - Mol. Cell Biol. Lipids* **2020**, *1865* (10), 158769.

(2) Pospelov, G.; Van Herck, W.; Burle, J.; Carmona Loaiza, J. M.; Durniak, C.; Fisher, J. M.; Ganeva, M.; Yurov, D.; Wuttke, J. BornAgain : Software for Simulating and Fitting Grazing-Incidence Small-Angle Scattering. *J. Appl. Crystallogr.* **2020**, *53* (1), 262–276.

(3) Campbell, R. A.; Wacklin, H. P.; Sutton, I.; Cubitt, R.; Fragneto, G. FIGARO: The New Horizontal Neutron Reflectometer at the ILL. *Eur. Phys. J. Plus* **2011**, *126* (11), 107.

(4) Clifton, L. A.; Paracini, N.; Hughes, A. V; Lakey, J. H.; Steinke, N.-J.; Cooper, J. F. K.; Gavutis, M.; Skoda, M. W. A. Self-Assembled Fluid Phase Floating Membranes with Tunable Water Interlayers. *Langmuir* **2019**, *35* (42), 13735–13744.

(5) Hafner, A.; Gutfreund, P.; Toperverg, B. P.; Jones, A. O. F.; de Silva, J. P.; Wildes, A.; Fischer, H. E.; Geoghegan, M.; Sferrazza, M. Combined Specular and Off-Specular Reflectometry: Elucidating the Complex Structure of Soft Buried Interfaces. *J. Appl. Crystallogr.* **2021**, *54* (3), 924–948.

Supplementary Figures:

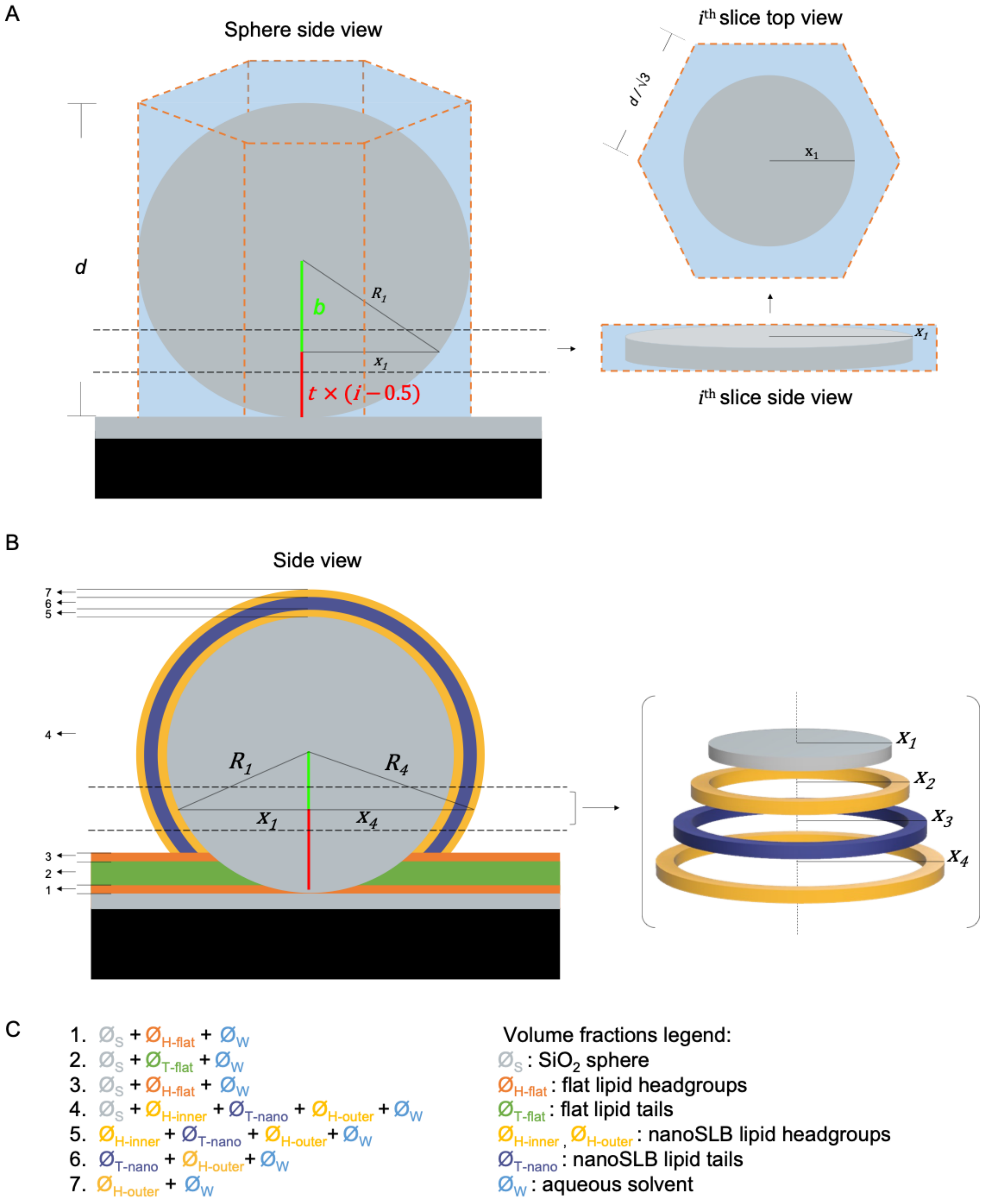

Figure S1 Schematic view of the model used to parametrise the nanoSLB array for the neutron reflectometry analysis (**A**) Side view of a silicon oxide nanoparticle enclosed in a hexagonal prism box (orange dashed line) backfilled with aqueous solvent (light blue). One the right a schematic representation of the ith slice shown. (**B**) Cartoon of the lipid-coated NP-array showing the seven regions with different components contributing to the overall SLD of each slice contained in the different regions.

Dimensions are not drawn to scale (**C**) Colour coded volume fractions composition of each one of the seven regions shown in **B** (left) and legend for each different volume fraction component (right)

A

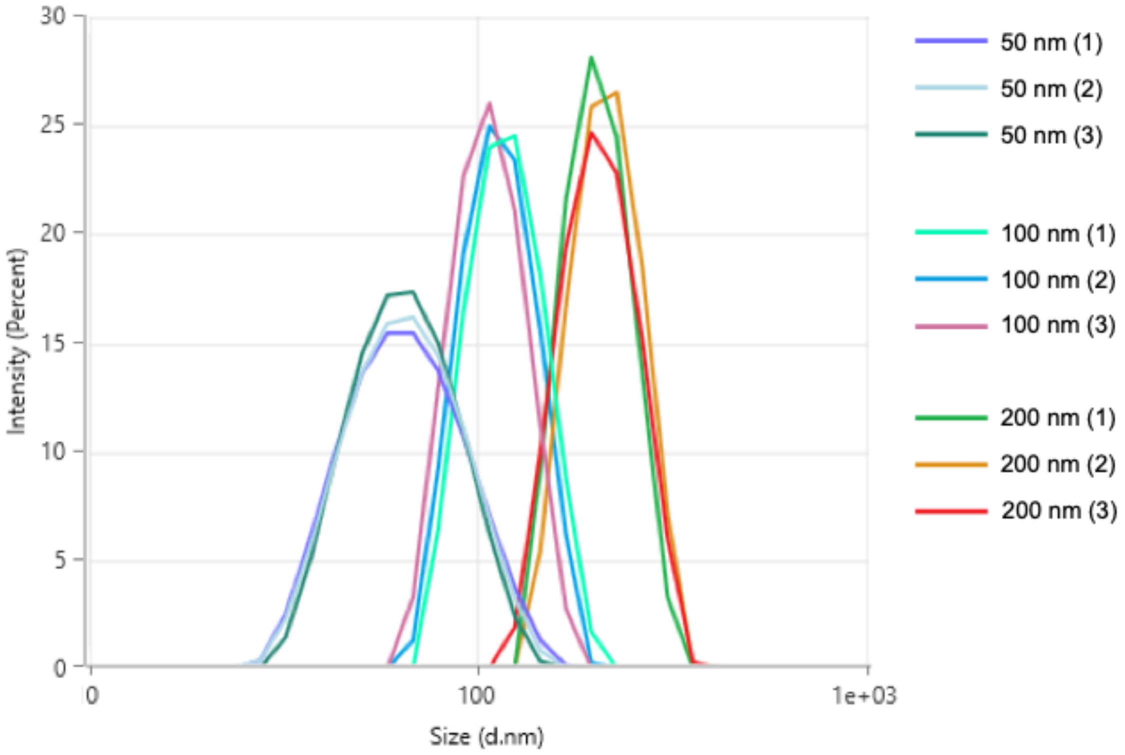

B

Nominal size	50 nm	100 nm	200 nm
DLS size (nm)	61.6 ± 0.5	111.0 ± 5.9	207.0 ± 6.9
PDI	0.097 ± 0.014	0.021 ± 0.001	0.041 ± 0.018

Figure S2 Dynamic light scattering (**A**) Size distribution of SiO_2 nanoparticles, triplicate measurements shown. The legend shows the nominal size of the commercial nanoparticles (**B**) Average and standard deviation of the size and polydispersity index obtained from each group of the three measurements shown in **A**

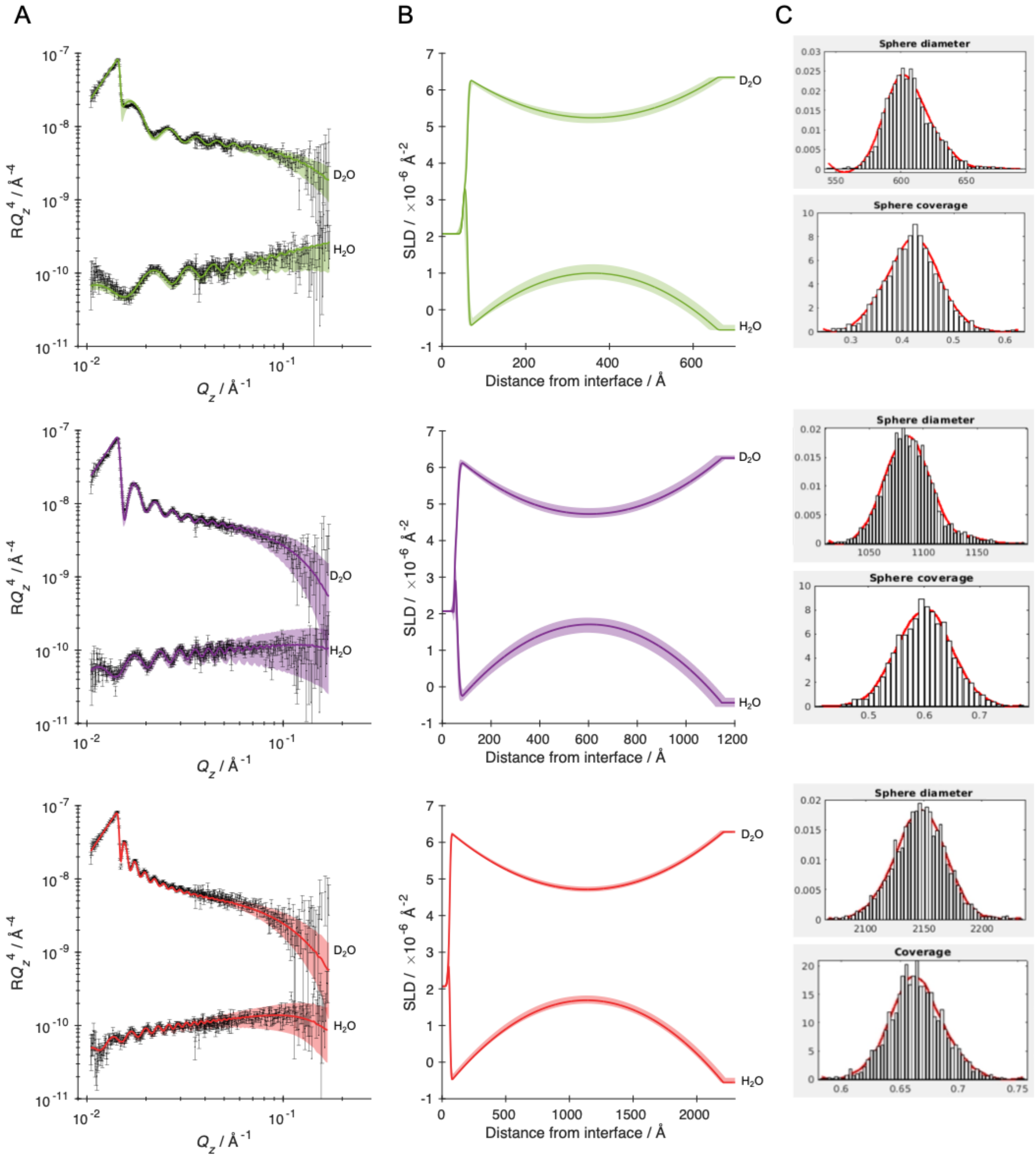

Figure S3 Error analysis of the reflectometry data for the bare SiO_2 nanoparticles (**A**) NR data (points), best fit (lines) and 65% confidence intervals (shades) for NP arrays measured in D_2O and H_2O. (**B**) corresponding SLD profiles (lines) and 65% confidence intervals (shades) obtained from the fit in **A**. (**C**) posterior distributions of the spheres diameter and coverage parameters obtained from the Bayesian error analysis for particles with nominal diameter of 50 nm (top), 100 nm (middle) and 200 nm (bottom)

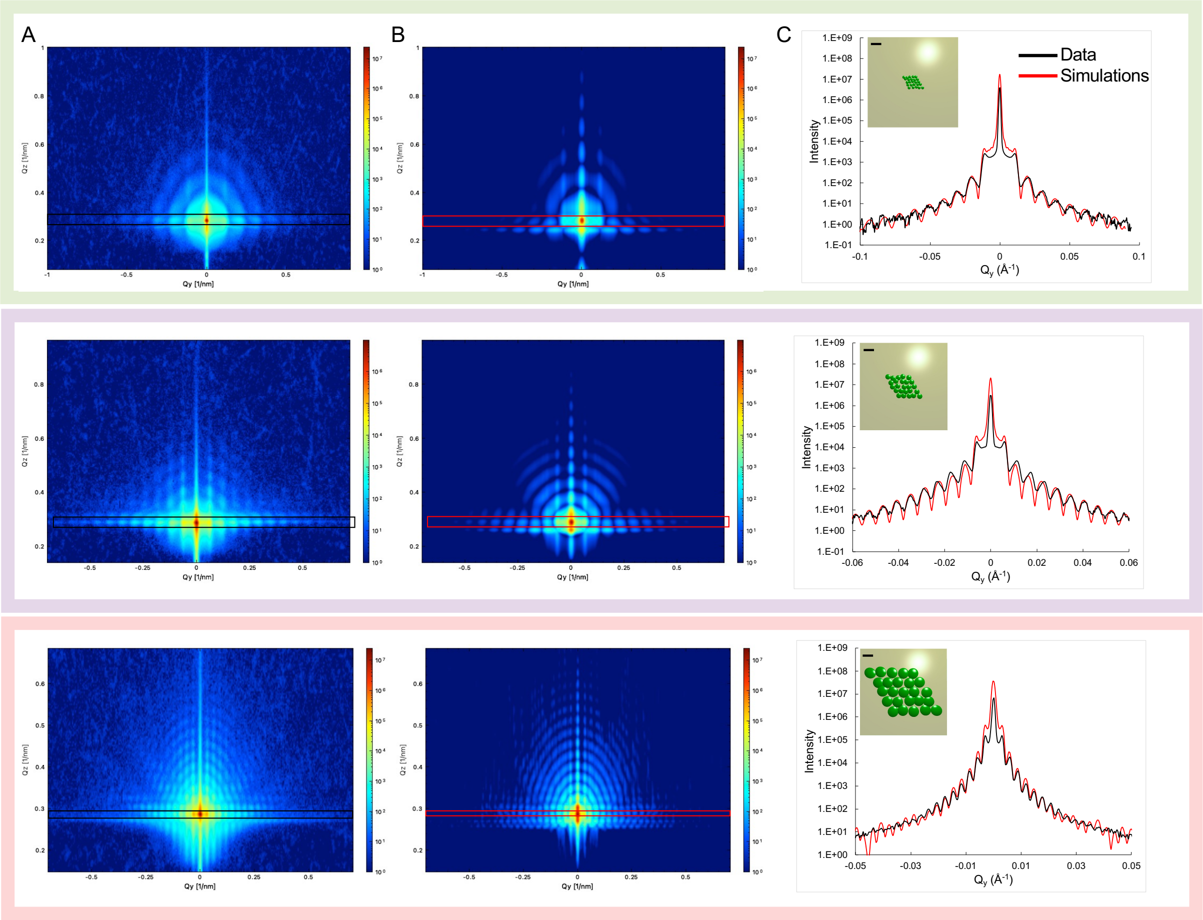

Figure S4 GISAXS simulations of NP monolayers at the air/solid interface (**A**) GISAXS data, (**B**) calculated signal from a NP monolayer using BornAgain, (**C**) comparison of the Q_y signal integrated at the specular angle for 50 nm (top), 100 nm (middle) and 200 nm NP (bottom). There is a common overestimation in intensity around the region close to the specular reflection in all cases. The intensities, the decay and the spacing of the fringes is reproduced for the three sizes. The model used in BornAgain simulates a finite 2D lattice composed of hexagonally packed SiO_2 spheres on a silicon substrate. The radius of the sphere was fixed to the monolayer thickness measured by neutron reflectometry and the lattice distances estimated in the software by manually varying the parameters until a reasonable agreement with the data was found. The radius of the spheres and the lattice distances estimated in the software together with the other parameters are given in **Table S2**.

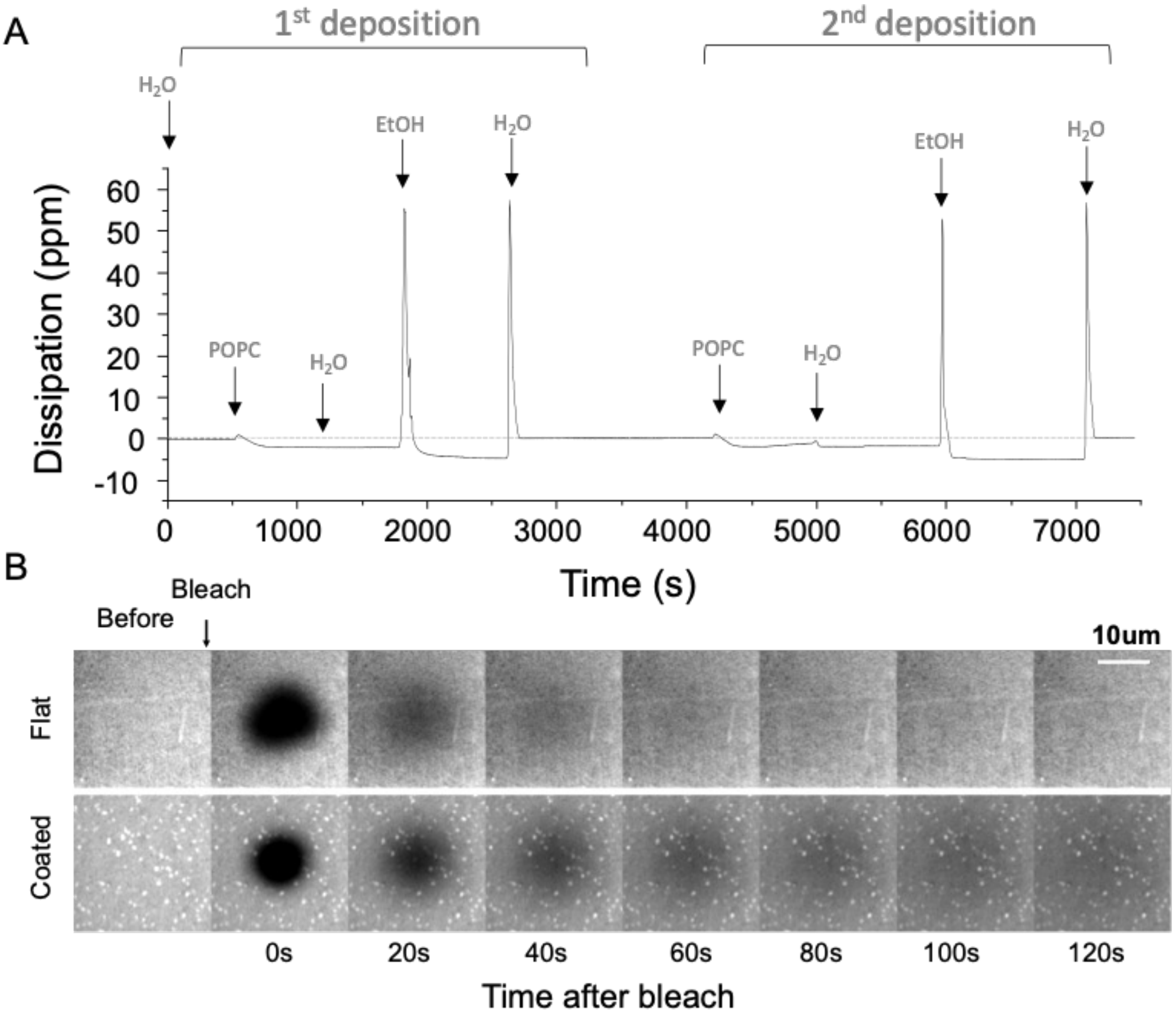

Figure S5 Supplementary information on QCMD and fluorescence measurements. (**A**) Dissipation trace of the deposition of the POPC bilayer on QCMD sensors coated with 200 nm SiNP. The dissipation shown here is the signal measured in the experiment shown in Figure 3 in the main text (**B**) Example of one of the fluorescence recovery images used in the FRAP experiments. Nanoparticles used were the 200 nm nominal diameter.

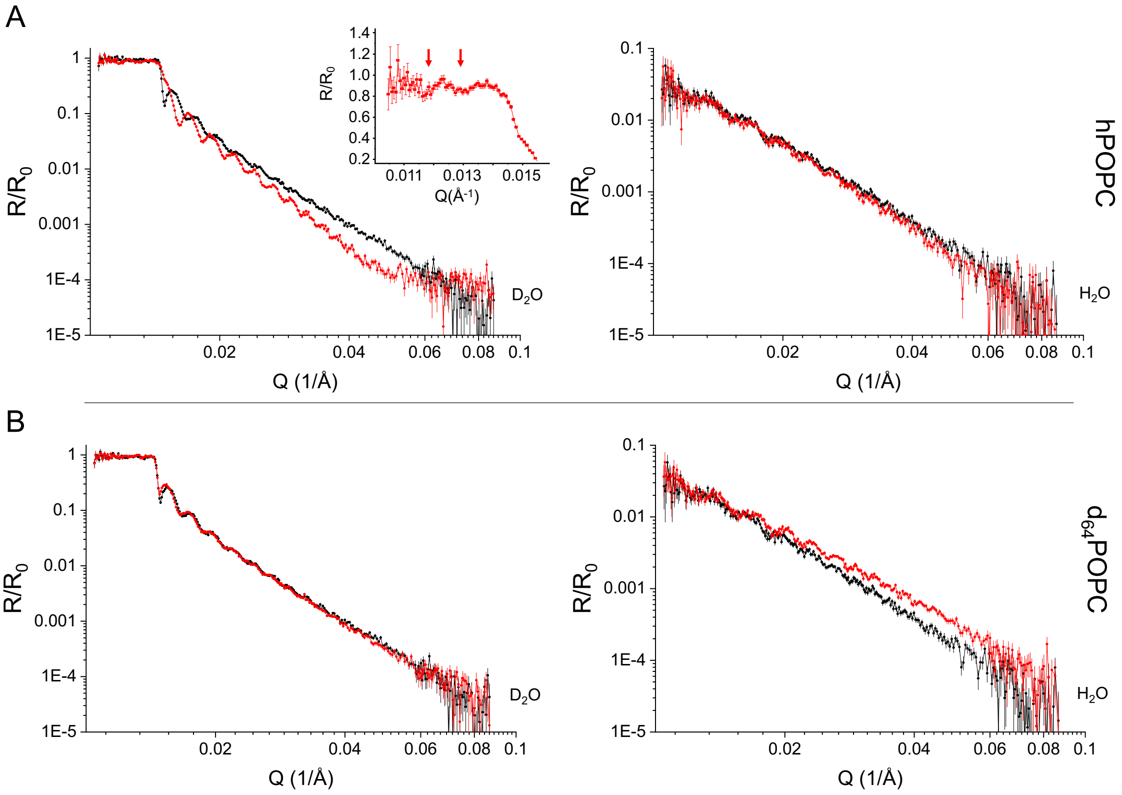

Figure S6 Effect of lipid addition on reflectivity profiles at different isotopic contrasts NR data before (black) and after (red) lipid addition to the NP array in D_2O (left) and H_2O (right). Curves for (**A**) hydrogenous POPC and (**B**) tail deuterated POPC. The inset shows the intensity dips due to the resonances below the critical Q (dashed line) of the reflectivity after the addition of d_{64}POPC in D_2O.

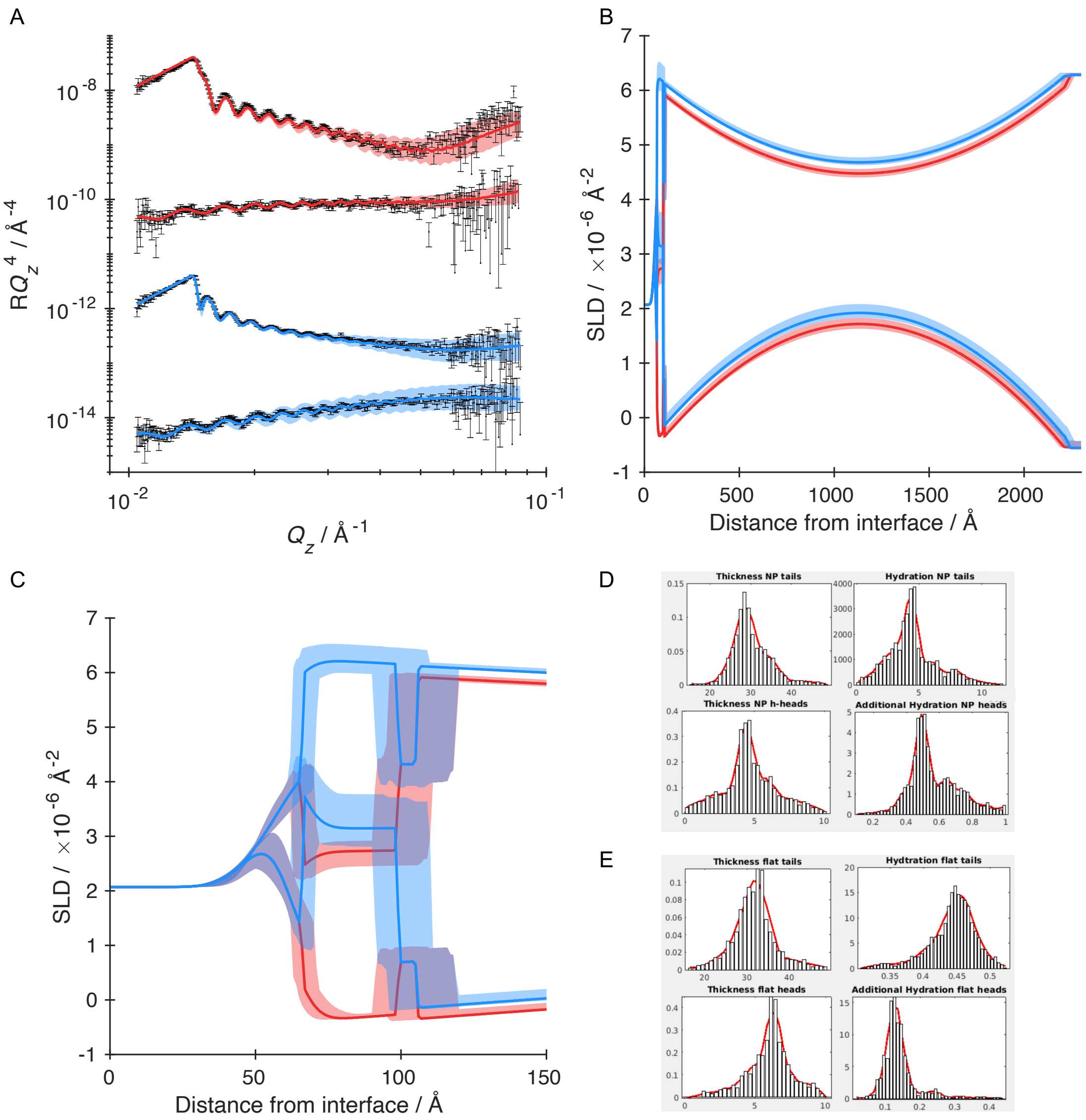

Figure S7 Error analysis of the reflectometry data for the nanoSLB (**A**) NR data (points), best fit (lines) and 65% confidence intervals (shades) for hydrogenous and deuterated nanoSLB arrays measured in D_2O and H_2O. (**B**) corresponding SLD profiles (lines) and 65% confidence intervals (shades) obtained from the fits shown in **A**. (**C**) Magnification of the region of the underlying planar bilayer shown in **B** (**D**) Posterior distributions of the parameters describing the nanoSLB and (**E**) posterior distributions of the parameters describing the underlying planar bilayer obtained from the Bayesian error analysis of the fits shown in **A.** Fit of the corresponding bare NP array are shown in **Figure S3**

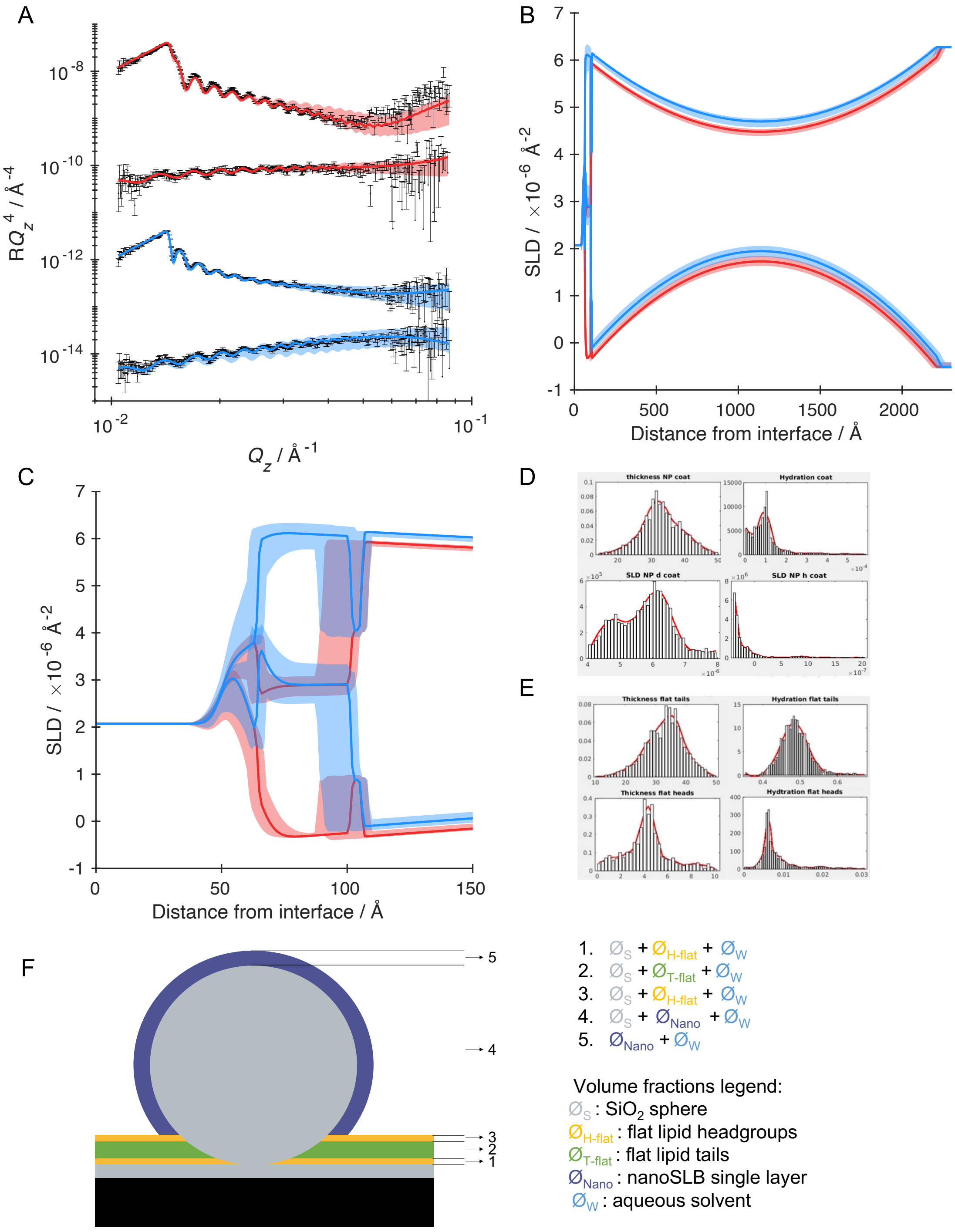

Figure S8 Error analysis of the reflectometry data for the nanoSLB modelled as a single uniform layer (**A**) NR data (points), best fit (lines) and 65% confidence intervals (shades) for hydrogenous and deuterated nanoSLB arrays measured in D_2O and H_2O. (**B**) corresponding SLD profiles (lines) and 65% confidence intervals (shades) obtained from the fits shown in **A**. (**C**) Magnification of the region of the underlying planar bilayer shown in **B** (**D**) Posterior distributions of the parameters describing the thickness, hydration and SLD of the hydrogenous and deuterated nanoSLB (**E**) Posterior distributions of the parameters describing the underlying planar bilayer. **D** and **E** are obtained from the Bayesian error analysis of the fits shown in **A**. (**F**) Schematic representation of the 5 regions required to model the nanoSLB using a single uniform layer and respective volume fractions of the component within each region

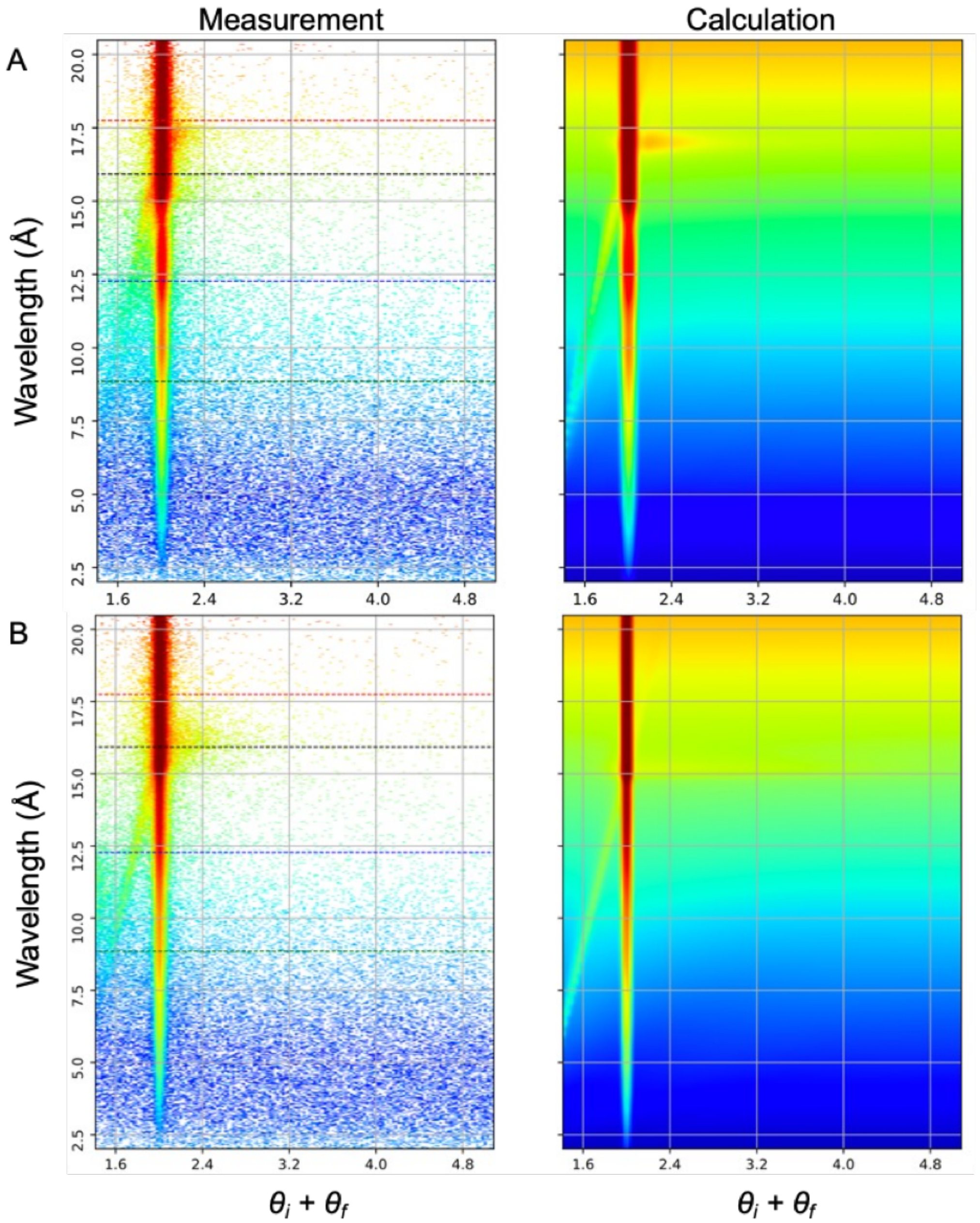

Figure S9 Measured and simulated off-specular signals Off-specular reflectometry signals (left) and simulated image (right) for (**A**) 100 nm and (**B**) 50 nm NP monolayers in D_2O.

Supplementary Tables:

Table S1 Parameters, best fit values, 65% confidence intervals (CI) and allowed fitting ranges relative to the fits of the bare SiO_2 NP arrays shown in Figure S3

	50 nm			100 nm			200 nm		
**Parameter**	**Estimated value**	**65% CI**	**Range**	**Estimated value**	**65% CI**	**Range**	**Estimated value**	**65% CI**	**Range**
Substrate roughness (Å)	3	[3 – 7]	[0 – 12]	2	[2 – 8]	[0 – 12]	9	[7 – 11]	[0 – 12]
SiO_2 thickness (Å)	11	[7 – 13]	[0 – 20]	12	[8 – 15]	[0 – 20]	12	[9 – 15]	[0 – 20]
SiO_2 roughness (Å)	3	[3 – 5]	[0 – 12]	8	[6 – 9]	[0 – 12]	7	[4 – 9]	[0 – 12]
Sphere diameter (Å)	602	[583 – 614]	[400 – 800]	1088	[1063 – 1102]	[800 – 1200]	2146	[2114 – 2179]	[1800 – 2400]
Sphere coverage (%)	42	[38 – 47]	[0 – 100]	61	[55 – 64]	[0 – 100]	66	[63 – 69]	[0 – 100]
SiO_2 SLD ($Å^{-2}$)	3.47e-6[a]	-		3.47e-6[a]	-		3.47e-6[a]	-	
Silicon SLD ($Å^{-2}$)	2.07e-6[a]	-		2.07e-6[a]	-		2.07e-6[a]	-	

[a] Parameters fixed to the calculated values

Table S2 Input for the simulations of the GISAXS signals shown in Figure S4

	50 nm	100 nm	200 nm
**Parameter**			
Diameter (Å)	600	1060	2140
Lattice distance (Å)	620	1100	2190
Lattice size	5x5	5x5	5x5
Particle density	0.65	0.72	0.81
SiO_2 SLD	1.88e-5	1.88e-5	1.88e-5
Silicon SLD	2.00e-5	2.00e-5	2.00e-5

Table S3 Parameters, best fit values, 65% confidence intervals (CI) and allowed fitting ranges relative to the fits of the nanoSLB shown in Figure S7 Values relative to the 200nm SiO_2 nanoparticles and the substrate parameters are shown in **Table S1**

	Parameter	Value	65% CI	Ranges
**Shared by both bilayers**	POPC heads SLD ($Å^{-2}$)	1.98e-6[a]	-	
	hPOPC tails SLD ($Å^{-2}$)	-3.0e-7[a]	-	
	dPOPC tails SLD ($Å^{-2}$)	6.3e-6	[5.9e-6 – 6.8e-6]	[5e-6 – 8e-6]
**Planar SLB**	POPC tails thickness (Å)	32	[25 – 37]	[0 – 50]
	POPC tails hydration (%)	45	[43 – 48]	[0 – 100]
	POPC headgroups thickness (Å)	7	[5 – 8]	[0 – 10]
	POPC additional heads hydration (%)	10[b]	[9 – 15]	[0 – 100]
**NanoSLB**	POPC tails thickness (Å)	29	[25 – 32]	[0 – 50]
	POPC tails hydration (%)	0.04	[0.01 – 0.05]	[0 – 100]
	POPC headgroups thickness (Å)	4	[3 – 6]	[0 – 10]
	POPC additional heads hydration (%)	49[b]	[42 – 67]	[0 – 100]

[a] Parameters fixed to the calculated values
[b] Total hydration of the headgroup region is given by the sum of tails and the additional headgroup hydration values

Table S4 Parameters, best fit values, 65% confidence intervals (CI) and allowed fitting ranges for the fits shown in Figure S8 relative to the model that describes the nanoSLB as a single uniform layer

	Parameter	Value	65% CI	Ranges
**Planar SLB**	POPC heads SLD ($Å^{-2}$)	1.98e-6[a]	-	
	hPOPC tails SLD ($Å^{-2}$)	-3.0e-7[a]	-	
	dPOPC tails SLD ($Å^{-2}$)	6.1e-6	[5.5e-6 – 6.5e-6]	[5e-6 – 8e-6]
	POPC tails thickness (Å)	36	[25 – 40]	[0 – 50]
	POPC tails hydration (%)	48	[45 – 51]	[0 – 100]
	POPC headgroups thickness (Å)	4	[2 – 5]	[0 – 10]
	POPC additional heads hydration (%)	6e-3[b]	[3e-3 – 8e-3]	[0 – 100]
**NanoSLB**	hCoat SLD	-3.8e-7	[-3.9e-7 – 2e-7]	[-3.9e-7 – 1e-6]
	dCoat SLD	6.15e-6	[4.4e-6 – 6.2e-6]	[4e-6 – 8e-6]
	Coat thickness (Å)	32	[25 – 32]	[0 – 50]
	Coat hydration (%)	9e-3	[4e-3 – 1e-2]	[0 – 100]

[a] Parameters fixed to the calculated values
[b] Total hydration of the headgroup region is given by the sum of tails and the additional headgroup hydration values
